# How human–AI feedback loops alter human perceptual, emotional and social judgements

**DOI:** 10.1038/s41562-024-02077-2

**Published:** 2024-12-18

**Authors:** Moshe Glickman, Tali Sharot

**Affiliations:** 1 Affective Brain Lab, Department of Experimental Psychology, University College London, London, UK; 2 Max Planck UCL Centre for Computational Psychiatry and Ageing Research, University College London, London, UK; 3 Department of Brain and Cognitive Sciences, Massachusetts Institute of Technology, Cambridge, MA USA

**Keywords:** Human behaviour, Decision making, Psychology

## Abstract

Artificial intelligence (AI) technologies are rapidly advancing, enhancing human capabilities across various fields spanning from finance to medicine. Despite their numerous advantages, AI systems can exhibit biased judgements in domains ranging from perception to emotion. Here, in a series of experiments (*n* = 1,401 participants), we reveal a feedback loop where human–AI interactions alter processes underlying human perceptual, emotional and social judgements, subsequently amplifying biases in humans. This amplification is significantly greater than that observed in interactions between humans, due to both the tendency of AI systems to amplify biases and the way humans perceive AI systems. Participants are often unaware of the extent of the AI’s influence, rendering them more susceptible to it. These findings uncover a mechanism wherein AI systems amplify biases, which are further internalized by humans, triggering a snowball effect where small errors in judgement escalate into much larger ones.

## Abstract (web summary)

Glickman and Sharot reveal a human–AI feedback loop, where AI amplifies subtle human biases, which are then further internalized by humans. This cycle, observed across various domains, leads to substantial increases in human bias over time.

## Main

Interactions between humans and artificial intelligence (AI) systems have become prevalent, transforming modern society at an unprecedented pace. A vital research challenge is to establish how these interactions alter human beliefs. While decades of research have characterized how humans influence each other^1–3^, the influence of AI on humans may be qualitatively and quantitatively different. This is partially because AI judgements are distinct from human judgements in several ways (for example, they tend to be less noisy^4^) and because humans may perceive AI judgements differently from those of other humans^5,6^. In this Article, we show how human**–**AI interactions impact human cognition. In particular, we reveal that when humans repeatedly interact with biased AI systems, they learn to be more biased themselves. We show this in a range of domains and algorithms, including a widely used real-world text-to-image AI system.

Modern AI systems rely on machine learning algorithms, such as convolutional neural networks^7^ (CNNs) and transformers^8^, to identify complex patterns in vast datasets, without requiring extensive explicit programming. These systems clearly augment human natural capabilities in a variety of domains, such as health care^9–11^, education^12^, marketing^13^ and finance^14^. However, it is well documented that AI systems can automate and perpetuate existing human biases in areas ranging from medical diagnoses to hiring decisions^15–17^, and may even amplify those biases^18–20^. While this problem has been established, a potentially more profound and complex concern has been largely overlooked until now. As critical decisions increasingly involve collaboration between AI and humans (for example, AI systems assisting physicians in diagnosis and offering humans advice on various topics^21,22^), these interactions provide a mechanism through which not only biased humans generate biased AI systems, but biased AI systems can alter human beliefs, leaving them more biased than they initially were. This possibility, predicted from a synthesis of bias amplification and human feedback learning, holds substantial implications for our modern society, but has not yet been empirically tested.

Bias, defined as a systematic error in judgements, can emerge in AI systems primarily due to inherent human biases embedded in the datasets the algorithm was trained on (‘bias in bias out’^23^; see also ref. ^24^) and/or when the data are more representative of one class than the other^25–27^. For example, generative AI systems such as text-to-image technologies and large language models learn from available data on the Internet, which being generated by humans contains inaccuracies and biases, even in cases where the ground truth exists. As a result, these AI systems end up reflecting a host of human biases (such as cognitive biases^28,29^, as well as racial and gender biases^30^). When humans subsequently interact with these systems (for example, by generating images or text), they may learn from them in turn. Interaction with other AI technologies that exhibit bias (including social bias), such as CNN-based facial recognition algorithms^31^, recommendation systems^32^, hiring tools^33^ and credit allocation tools^34^, may also induce similar circularity. Moreover, human biases can be amplified even when individuals are not directly interacting with an AI system, but merely observing its output. Indeed, an estimated 15 billion AI-generated images circulate online^35^, which users routinely consume passively on social media, news websites and other digital platforms. As a result, the impact of AI-generated content on human biases may extend beyond the immediate users of these systems.

Here, over a series of studies, we demonstrate that when humans and AI interact, even minute perceptual, emotional and social biases originating either from AI systems or humans leave human beliefs more biased, potentially forming a feedback loop. The impact of AI on humans’ beliefs is gradually observed over time, as humans slowly learn from the AI systems. We uncover that the amplification effect is greater in human**–**AI interactions than in human**–**human interactions, due both to human perception of AI and the unique characteristics of AI judgements. In particular, AI systems may be more sensitive to minor biases in the data than humans due to their expansive computational resources^36^ and may therefore be more likely to leverage them to improve prediction accuracy, especially when the data are noisy^37^. Moreover, once trained, AI systems’ judgements tend to be less noisy than those of humans^4^. Thus, AI systems provide a higher signal-to-noise ratio than humans, which enables rapid learning by humans, even if the signal is biased. In fact, if the AI is perceived as being superior to humans^6,38,39^ (but see ref. ^40^), learning its bias can be considered perfectly rational. Amplification of bias only occurs if the bias already exists in the system: when humans interact with an accurate AI system, their judgements are improved.

## Results

### Human–AI feedback loops can amplify human’s biases

**Fig. 1:**
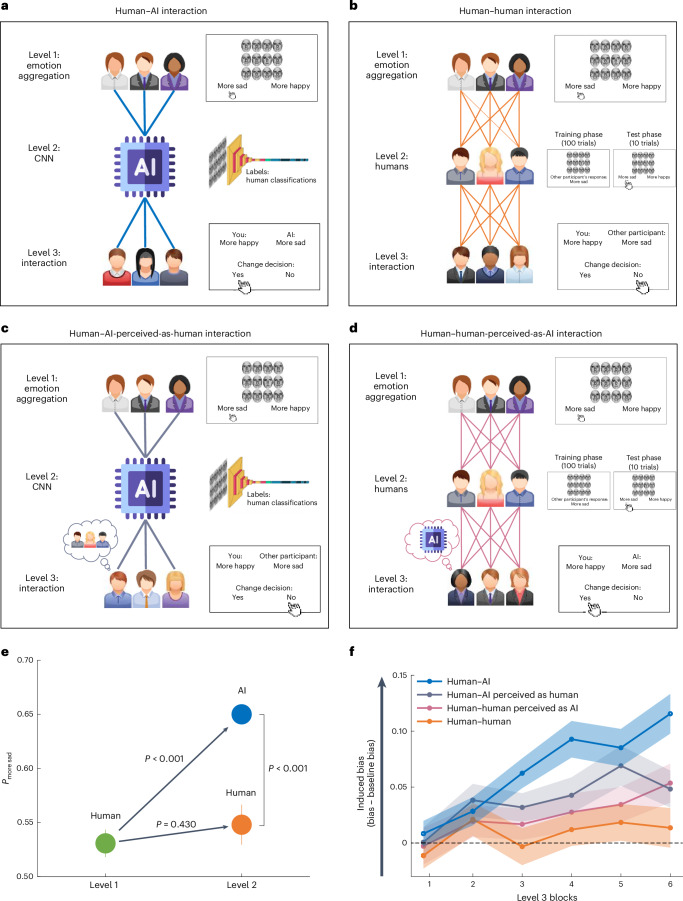
Human**–**AI interaction creates a feedback loop that makes humans more biased (experiment 1). **a,** Human**–**AI interaction. Human classifications in an emotion aggregation task are collected (level 1) and fed to an AI algorithm (CNN; level 2). A new pool of human participants (level 3) then interact with the AI. During level 1 (emotion aggregation), participants are presented with an array of 12 faces and asked to classify the mean emotion expressed by the faces as more sad or more happy. During level 2 (CNN), the CNN is trained on human data from level 1. During level 3 (human**–**AI interaction), a new group of participants provide their emotion aggregation response and are then presented with the response of an AI before being asked whether they would like to change their initial response. **b**, Human–human interaction. This is conceptually similar to the human–AI interaction, except the AI (level 2) is replaced with human participants. The participants in level 2 are presented with the arrays and responses of the participants in level 1 (training phase) and then judge new arrays on their own as either more sad or more happy (test phase). The participants in level 3 are then presented with the responses of the human participants from level 2 and asked whether they would like to change their initial response. **c**, Human**–**AI-perceived-as-human interaction. This condition is also conceptually similar to the human**–**AI interaction condition, except participants in level 3 are told they are interacting with another human when in fact they are interacting with an AI system (input: AI; label: human). **d**, Human–human-perceived-as-AI interaction. This condition is similar to the human–human interaction condition, except that participants in level 3 are told they are interacting with AI when in fact they are interacting with other humans (input: human; label: AI). **e**, Level 1 and 2 results. Participants in level 1 (green circle; *n* = 50) showed a slight bias towards the response more sad. This bias was amplified by AI in level 2 (blue circle), but not by human participants in level 2 (orange circle; *n* = 50). The *P* values were derived using permutation tests. All significant *P* values remained significant after applying Benjamini–Hochberg false discovery rate correction at *α* = 0.05. **f**, Level 3 results. When interacting with the biased AI, participants became more biased over time (human–AI interaction; blue line). In contrast, no bias amplification was observed when interacting with humans (human–human interaction; orange line). When interacting with an AI labelled as human (human–AI-perceived-as-human interaction; grey line) or humans labelled as AI (human–AI-perceived-as-human interaction; pink line), participants’ bias increased but less than for the human–AI interaction (*n* = 200 participants). The shaded areas and error bars represent s.e.m.

We begin by collecting human data in an emotion aggregation task in which human judgement is slightly biased. We then demonstrate that training an AI algorithm on this slightly biased dataset results in the algorithm not only adopting the bias but further amplifying it. Next, we show that when humans interact with the biased AI, their initial bias increases (Fig. 1a; human**–**AI interaction). This bias amplification does not occur in an interaction including only human participants (Fig. 1b; human–human interaction).

#### Humans exhibit a small judgement bias

Fifty participants performed an emotion aggregation task (adapted from refs. ^41–44^). On each of 100 trials, participants were presented briefly (500 ms) with an array of 12 faces and were asked to report whether the mean emotion expressed by the faces in the array was more sad or more happy (Fig. 1a; level 1). The faces were sampled from a dataset of 50 morphed faces, created by linearly interpolating between sad and happy expressions (Methods). Based on the morphing ratio, each face was ranked from 1 (100% sad face) to 50 (100% happy face). These rankings were closely associated with participants’ own rankings of each face when observed one by one (*b* = 0.8; *t*(50) = 26.25; *P* < 0.001; see Supplementary Results). We created 100 unique arrays of 12 faces for each participant. The average ranking of the 12 faces in half of the arrays was smaller than 25.5 (thus, the array was more sad) and greater than 25.5 in the other half (thus the array was more happy).

Bias in this task was defined as the difference between the average responses of a participant across all trials and the actual average. The actual average in the task was 0.5, as responses were coded as either 1 (more sad) or 0 (more happy), and exactly half of the trials were more sad and half were more happy. Mathematically, the bias is expressed as:

$${{\rm{Bias}}}=\frac{1}{n}\mathop{\sum }\limits_{i=1}^{n}{C}_{i}-0.5$$

Where *n* denotes the total number of data points and *C*_*i*_ denotes the classification assigned to each data point (*C*_*i*_ = 1 for a more sad classification and *C*_*i*_ = 0 for a more happy classification). A positive bias indicates a tendency towards classifying responses as more sad, whereas a negative bias suggests a leaning towards classifying responses as more happy. For example, if a participant were to classify 0.7 of the arrays as more sad, their bias would be 0.7 − 0.5 = 0.2, whereas if they were to classify 0.3 of the arrays as more sad, their bias would be 0.3 − 0.5 = −0.2.

Consistent with previous studies showing that interpretation of an ambiguous valence is more likely to be negative under short encoding times^45,46^, participants showed a slight but significant tendency to report that the faces were more sad. In particular, they categorized 53.08% of the arrays as more sad, which is a greater proportion than would be expected by chance (permutation test against 50%: *P* = 0.017; *d* = 0.34; 95% confidence interval (CI)_more sad_ = 0.51 to 0.56; green circle in Fig. 1e; see also Supplementary Results for estimation of the bias by psychometric function analysis). The bias was much larger in the first block than subsequent blocks (*M*_block 1_ = 56.72%; *M*_blocks 2–4_ = 51.87%; permutation test comparing the first block with the rest: *P* = 0.002; *d* = 0.46; 95% CI = 0.02 to 0.08), suggesting that the participants corrected their bias over time.

#### AI trained on biased human data amplifies the bias

Next, we used a CNN^7^ to classify each array of faces into more happy or more sad. As detailed below, the CNN amplified the classification bias observed in the human participants (see Methods for further details of the model).

First, to test the accuracy of the model, we trained it on the 5,000 arrays that were presented to the participants in level 1 (5,000 arrays = 50 participants × 100 arrays), with class labels based on the objective ranking scores of the arrays (that is, not the human labels). The model was then evaluated on a 300 out-of-sample test set and showed a classification accuracy of 96%, suggesting that it was highly accurate and did not show a bias if trained on non-biased data (see Table 1). Next, we trained the model on class labels defined based on the human classification (5,000 samples of arrays; Fig. 1a) and evaluated it on 300 arrays in an out-of-sample test set. The model classified the average emotion as more sad in 65.33% of the cases, despite only 50% of the arrays being more sad. This number was significantly greater than would be expected by chance (permutation test against 50%: *P* < 0.001; 95% CI_more sad_ = 0.60 to 0.71; blue circle in Fig. 1e) and significantly greater than the bias observed in the human data (level 1), which was only 53% (permutation test: *P* < 0.001; *d* = 1.33; 95% CI = 0.09 to 0.14; Fig. 1e). In other words, the AI algorithm greatly amplified the human bias embedded in the data it was trained on. Similar results were obtained for CNNs with different architectures, including ResNet50 (ref. ^47^; see Supplementary Results).

**Table 1:** Accuracy and bias in the training data and CNN classifications

Labels	Objective ranking (accuracy = 100%; bias = 0%)	Objective ranking + minor bias (accuracy = 97%; bias = 3%)	Participant classifications (accuracy = 63%; bias = 3%)	Random labels + minor bias (accuracy = 50%; bias = 3%)
Accuracy − objective labels	96%	94%	66%	50%
Accuracy – training labels	96%	92%	69%	53%
Bias	1%	3%	15%	50%

Training was conducted using four different label sets: (1) objective (based on morphing ranking scores); (2) objective with a 3% bias; (3) participant classifications; and (4) random labels with a 3% bias. The predictions of the model were assessed on an out-of-sample test set of 300 arrays. Accuracy and bias were evaluated with respect to the objective labels and with respect to the labels the models were trained on (training labels).

A possible reason for the bias amplification of the AI is that it exploits biases in the data to improve its prediction accuracy. This should happen more when the data are noisy or inconsistent. To test this hypothesis, we retrained the model with two new sets of labels. First, we used non-noisy labels (that is, based on the objective ranking scores of the arrays), but induced a minor bias by switching 3% of the labels. Thus, 53% of the labels were classified as more sad. Second, we used very noisy labels (random labels), in which we also induced a 3% bias. If the bias amplification were due to noise, the bias of the latter model should be higher than that of the former. The results confirmed this hypothesis (Table 1): the average bias of the model trained on the accurate labels with a minor bias was exactly 3%, whereas the average bias of the model trained on the random labels with a bias of 3% was 50% (that is, the model classified 100% of arrays as more sad). These results indicate that the bias amplification of the CNN model is related to the noise in the data.

#### Interaction with biased AI increases human bias

Next, we set out to examine whether interacting with the biased AI algorithm would alter human judgements (Fig. 1a; level 3). To this end, we first measured participants’ baseline performance on the emotion aggregation task for 150 trials, so that we could compare their judgements after interacting with the AI versus before. As in level 1, we found that participants had a small bias at first (*M*_block 1_ = 52.23%), which decreased in subsequent blocks, (*M*_blocks 2–5_ = 49.23%; permutation test testing the first block against the rest of the blocks: *P* = 0.03; *d* = 0.31; 95% CI = 0.01 to 0.06). The next question was whether interacting with AI would cause the bias to reappear in humans and perhaps even increase.

To test this hypothesis, on each of 300 trials, participants first indicated whether the array of 12 faces was more sad or more happy. They were then presented with the response of the AI to the same array (participants were told that they “will be presented with the response of an AI algorithm that was trained to perform the task”). They were then asked whether they would like to change their initial response or not (that is, from more sad to more happy or vice versa). The participants changed their response on 32.72% (±2.3% s.e.) of the trials in which the AI provided a different response and on 0.3% (±0.1% s.e.) of the trials in which the AI provided the same response as they did (these proportions are significantly different: permutation test: *P* < 0.001; *d* = 1.97; 95% CI = 0.28 to 0.37). Further study (Supplementary Experiment 1) showed that when not interacting with any associate, participants changed their decisions only on 3.97% of trials, which was less than when interacting with a disagreeing AI (permutation test: *P* < 0.001; *d* = −2.53; 95% CI = −0.57 to −0.42) and more than when interacting with an agreeing AI (permutation test: *P* < 0.001; *d* = 0.98; 95% CI = 0.02 to 0.05).

The primary question of interest, however, was not whether participants changed their response after observing the AI’s response. Rather, it was whether over time their own response regarding an array (before observing the AI’s response to that specific array) became more and more biased due to previous interactions with the AI. That is, did participants learn to become more biased over time?

Indeed, whereas in the baseline blocks participants classified on average only 49.9% (±1.1% s.e.) of the arrays as more sad, when interacting with the AI this rate increased significantly to 56.3% (±1.1% s.e.; permutation test for interaction blocks against baseline: *P* < 0.001; *d* = 0.84; 95% CI_more sad_ = 0.54 to 0.59). The learned bias increased over time: in the first interaction block it was only 50.72%, whereas in the last interaction block it was 61.44%. This increase in bias was confirmed by a linear mixed model predicting a higher rate of more sad classifications as the block number (a fixed factor) increased, with random intercepts and slopes at the participant level (*b* = 0.02; *t*(50) = 6.23; *P* < 0.001; Fig. 1f).

These results demonstrate an algorithmic bias feedback loop; training an AI algorithm on a set of slightly biased human data results in the algorithm amplifying it. Subsequent interactions of other humans with this algorithm further increase the humans’ initial bias levels, creating a feedback loop.

### Human–human interactions did not amplify bias

Next, we investigated whether the same degree of bias contagion occurs in interactions involving only humans. To this end, we used the same interaction structure as above, except the AI system was replaced with human participants (Fig. 1b).

#### Humans exhibit a small judgement bias

The responses used in the first level of the human–human interaction were the same as those used in the human–AI interaction described above.

#### Humans trained on human data do not amplify bias

Conceptually similar to AI algorithm training, here we aimed to train humans on human data (Fig. 1b; level 2). The participants were presented with 100 arrays of 12 faces. They were told they would be presented with the responses of other participants who performed the task before. For each of the 100 arrays, they observed the response of a pseudo-randomly selected participant from level 1 (see Methods for further details). Thereafter, they judged ten new arrays on their own (as either more sad or more happy). To verify that the participants attended to the responses of the other level 1 participants, they were asked to report them on 20% of the trials (randomly chosen). Participants who gave an incorrect answer on more than 10% of the trials (and thus were not attending the task; *n* = 14), were excluded from the experiment.

Participants characterized the arrays as more sad 54.8% of the time, which is more than would be expected by chance (permutation test against 50%: *P* = 0.007; *d* = 0.41; 95% CI_more sad_ = 52 to 58%). Critically, this result did not differ from that of level 1 human participants (permutation test level 1 humans versus level 2 humans: *P* = 0.43; *d* = 0.11; 95% CI = −0.02 to 0.06; Fig. 1e), but was significantly lower than for the AI algorithm, which characterized 65.13% of the arrays as more sad (permutation test level 2 humans against level 2 AI: *P* < 0.001; *d* = 0.86; 95% CI = −0.07 to −0.013; Fig. 1e). This difference was unlikely to have been driven by variations in training sample sizes, as the effect was observed even when AI and human participants were trained on identical datasets (Supplementary Experiment 2). Furthermore, the results were generalized to a different training method, in which participants were incentivized to actively predict the responses of other participants (Supplementary Experiment 3).

In conclusion, unlike the AI, human bias was not amplified after being trained on biased human data. This is not surprising, as the level of bias participants in level 2 naturally exhibit is probably the same as the one they were trained on. Moreover, unlike AI systems, humans base their judgements on factors that go beyond the training session, such as previous experiences and expectations.

#### Human–human interaction does not increase bias

Next, we exposed a new pool of participants (*n* = 50) to the judgements of humans from level 2. The task and analysis were identical to those described for level 3 of the human–AI interaction (except, of course, participants were interacting with humans, which they were made aware of; Fig. 1b).

Before being exposed to the other human’s response, participants completed five baseline blocks. As in levels 1 and 3 (human–AI interaction), participants showed a significant bias during the first block *(M*_block 1_ = 53.67%) which disappeared over time (*M*_blocks 2–5_ = 49.87%; permutation test for the first baseline block against the rest of the baseline blocks: *P* = 0.007; *d* = 0.40; 95% CI = 0.01 to 0.06).

Next, participants interacted with other human participants (human–human interaction; level 2). As expected, participants changed their classification more when the other participants disagreed with them (11.27 ± 1.4% s.e.) than when they agreed with them (0.2 ± 0.03% s.e.) (permutation test comparing the two: *P* < 0.001; *d* = 1.11; 95% CI = 0.08 to 0.14) and less than when interacting with a disagreeing AI (which was 32.72%; permutation test comparing the response change when interacting with a disagreeing AI compared with interacting with a disagreeing human: *P* < 0.001; *d* = 1.07; 95% CI = 0.16 to 0.27).

Importantly, there was no evidence of learned bias in the human–human interaction (Fig. 1f). Classification rates were no different when interacting with other humans (*M*_more sad_ = 51.45 ± 1.3% s.e.) than baseline (50.6 ± 1.3% s.e.) (permutation test for interaction blocks against baseline: *P* = 0.48; *d* = 0.10; 95% CI_more sad_ = −0.01 to 0.03) and did not change over time (*b* = 0.003; *t*(50) = 1.1; *P* = 0.27).

Taken together, these results indicate that human bias is significantly amplified in a human–AI interaction, more so than in interactions between humans. These findings suggest that the impact of biased AI systems extends beyond their own biased judgement to their ability to bias human judgement. This raises concerns for human interactions with potentially biased algorithms across different domains.

#### AI’s output and human perception of AI shape its influence

A question that arises is whether participants became more biased when interacting with the AI system compared with humans because the AI provided more biased judgements, because they perceived the AI system differently than other humans, or both. To address this question, we ran two additional iterations of the experiment. In the first iteration (AI perceived as human), participants interacted with an AI system but were told they were interacting with another human participant (Fig. 1c). In the second iteration (human perceived as AI), participants interacted with an AI system but were told they were interacting with another human participant (Fig. 1d).

To this end, new pools of participants (*n* = 50 per condition) were recruited. First, they performed the baseline test described above and then they interacted with their associate (level 3). When interacting with the AI (which was believed to be a human) participants’ bias increased over time: in the first interaction block it was only 50.5%, whereas in the last interaction block it was 55.28% (Fig. 1f). The increase in bias across blocks was confirmed by a linear mixed model predicting a higher rate of more sad classifications as the block number (a fixed factor) increased, with random intercepts and slopes at the participant level (*b* = 0.01; *t*(50) = 3.14; *P* < 0.001). Similar results were obtained for the human–human-perceived-as-AI interaction. The bias increased across blocks (from 49.0% in the first block to 54.6% in the last), as was confirmed by a linear mixed model (*b* = 0.01; *t*(50) = 2.85; *P* = 0.004; Fig. 1f). In both cases, the bias was greater than at baseline (human–AI perceived as human: *M*_bias_ = 3.85 (permutation test comparing with baseline: *P* = 0.001; *d* = 0.49; 95% CI = 0.02 to 0.06); human–human perceived as AI: *M*_bias_ = 2.49 (permutation test comparing with baseline: *P* = 0.04; *d* = 0.29; 95% CI = 0.01 to 0.05)).

Was the induced bias a consequence of the type of input (AI versus human) or the perception of that input (perceived as AI versus perceived as human)? To investigate this, we submitted the induced bias scores (the percentage of more sad judgements minus the baseline percentage of more sad judgements) into a 2 (input: AI versus human) × 2 (label: AI versus human) analysis of variance (ANOVA) with time (blocks 1–6) as a covariate (Fig. 1f). The results revealed interactions between input and time (*F*(4.55, 892.35) = 3.40; *P* = 0.006) and between label and time (*F*(4.55, 892.35) = 2.65; *P* = 0.026). In addition, there were main effects of input (*F*(1, 196) = 9.45; *P* = 0.002) and time (*F*(4.55, 892.35) = 14.80; *P* < 0.001). No other effects were significant (all *P* values > 0.06). Thus, as illustrated in Fig. 1f, both the AI’s input and its label contributed to enhanced bias in humans over time.

Finally, we assessed the rate of decision changes among participants. Participants were more likely to change their classification when their associate disagreed with them. In human–AI-perceived-as-human interactions, decision changes occurred at a rate of 16.84% (±1.2% s.e.) when there was a disagreement, compared with a mere 0.2% (±0.05% s.e.) when agreeing (permutation test comparing the two: *P* < 0.001; *d* = 1.22; 95% CI = 0.13 to 0.20). Similarly, for the human–human-perceived-as-AI condition, decision changes were observed in 31.84% (±2.5% s.e.) when disagreement existed, compared with 0.4% (±0.1% s.e.) in cases of agreement (permutation test comparing the two: *P* < 0.001; *d* = 1.7; 95% CI = 0.26 to 0.36).

To quantify the effects of input and label on decision changes in cases of disagreement, we submitted the percentage of decision change into a 2 (input: AI versus human) × 2 (label: AI versus human) ANOVA with time (blocks 1–6) as a covariate. The results revealed that both the AI’s input (*F*(1, 196) = 7.05; *P* = 0.009) and its label (*F*(1, 196) = 76.30; *P* < 0.001) increased the likelihood of a decision change. These results remained consistent after applying Welch’s correction to address violations of the homogeneity of variance assumption: for AI’s input *F*(1, 197.92) = 5.11 and *P* = 0.02 and for AI’s label *F*(1, 175.57) = 74.21 and *P* < 0.001. All other main effects and interactions were not significant (all *P* values > 0.13).

### Biased algorithms bias decisions, whereas accurate ones improve them

**Fig. 2:**
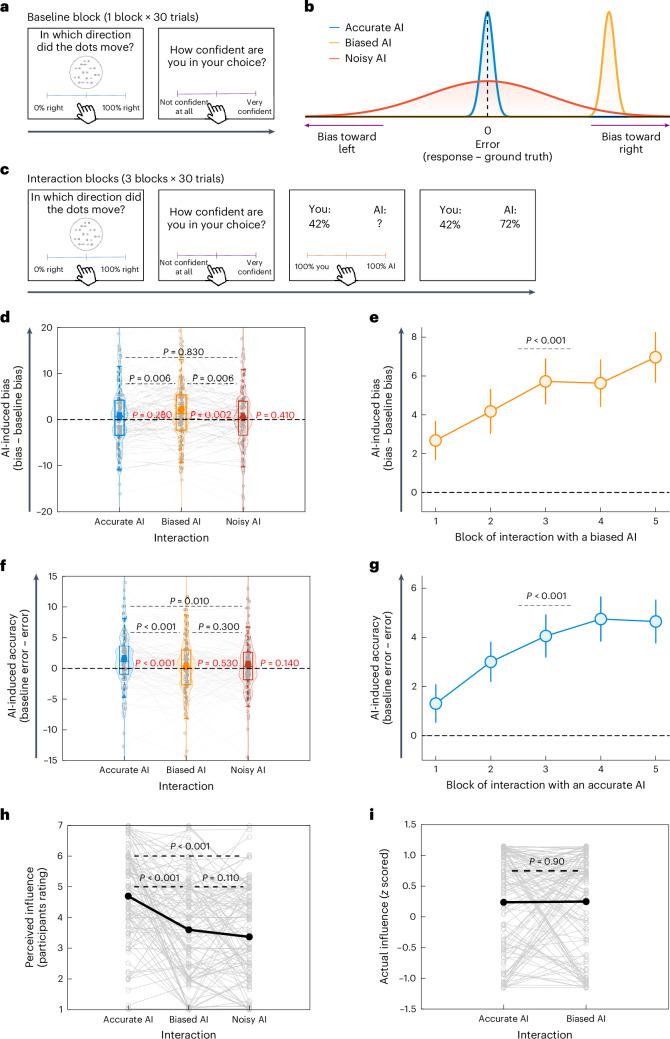
A biased algorithm produces human bias, whereas an accurate algorithm improves human judgement. **a**, Baseline block. Participants performed the RDK task, in which an array of moving dots was presented for 1 s. They estimated the percentage of dots that moved from left to right and reported their confidence. **b**, Algorithms. Participants interacted with three algorithms: accurate (blue distribution), biased (orange distribution) and noisy (red distribution). **c**, Interaction blocks. Participants provided their independent judgement and confidence (self-paced) and then observed their own response and a question mark where the AI algorithm response would later appear. Participants were asked to assign weights to their response and the response of the algorithm (self-paced). Thereafter, the response of the algorithm was revealed (2 s). Note that the AI algorithm’s response was revealed only after the participants indicated their weighting. As a result, they had to rely on their global evaluation of the AI based on previous trials. **d**, AI-induced bias. Interacting with a biased AI resulted in significant human bias relative to baseline (*P* values shown in red) and relative to interactions with the other algorithms (*P* values shown in black; *n* = 120). **e**, When interacting with a biased algorithm, AI-induced bias increases over time (*n* = 50). **f**, AI-induced accuracy change. Interacting with an accurate AI resulted in a significant increase in human accuracy (that is, reduced error) relative to baseline (*P* values shown in red) and relative to interactions with the other algorithms (*P* values shown in black; *n* = 120). **g**, When interacting with an accurate algorithm, AI-induced accuracy increases over time (*n* = 50). **h**,**i**, Participants perceived the influence of the accurate algorithm on their judgements to be greatest (**h**; *n* = 120), even though the actual influence of the accurate and biased algorithms was the same (**i**; *n* = 120). The thin grey lines and circles correspond to individual participants. In **d** and **f**, the circles correspond to group means, the central lines represent median values and the bottom and top edges are the 25th and 75th percentiles, respectively. In **e** and **g**, the error bars represent s.e.m. The *P* values were derived using permutation tests. All significant *P* values remained significant after applying Benjamini–Hochberg false discovery rate correction at *α* = 0.05.

Next, we sought to generalize the above results to different types of algorithm and domain. In particular, we aimed to mimic a situation in which humans are not a priori biased, but rather AI bias emerges for other reasons (for example, if it was trained on unbalanced data). To this end, we employed a variant of the random dot kinematogram (RDK) task^48–51^, in which participants were presented with an array of moving dots and asked to estimate the percentage of dots that moved from left to right on a scale ranging from 0% (no dots moved from left to right) to 100% (all dots moved from left to right). To estimate baseline performance, participants first performed the RDK task on their own for 30 trials and reported their confidence on a scale ranging from not confident at all to very confident (Fig. 2a). Across trials, the actual average percentage of dots that moved rightward was 50.13 ± 20.18% (s.d.), which was not significantly different from 50% (permutation test against 50%: *P* = 0.98; *d* = 0.01; 95% CI = 42.93 to 57.33%), and the average confidence was 0.56 ± 0.17 (s.d.).

To examine whether and how different algorithmic response patterns affect human decision-making, we used three simple algorithms: accurate, biased and noisy. The accurate algorithm always indicated the correct percentage of dots that moved from left to right (Fig. 2b; blue distribution). The biased algorithm provided systematically upward biased estimates of dots that moved to the right (Fig. 2b; orange distribution; *M*_bias_ = 24.96). The noisy algorithm provided responses that were equal to those of the accurate algorithm plus Gaussian noise (s.d. = 30; Fig. 2b; red distribution). The biased and noisy algorithms had the same absolute error (Methods). The algorithms used here were hard coded to allow full control over their responses.

On each trial, participants first provided their judgement and confidence and then observed their own response and a question mark where the algorithm response would later appear (Fig. 2c). They were asked to assign weight to their own response and to that of the algorithm on a scale ranging from 100% you to 100% AI (Methods). Thus, if a participant assigned a weight of *w* to their own response, the final joint decision would be:

$$\begin{array}{l}{{\rm{Final}}\; {\rm{joint}}\; {\rm{decision}}}\\=w \times({{\rm{participant}}}\mbox{'}{{\rm{s}}\; {\rm{response}}})+(1-w)\times\,({{\rm{AI}}}\mbox{'}{{\rm{s}}\; {\rm{response}}})\end{array}$$

This weighting task is analogous to the change decision task in experiment 1; however, here we used a continuous scale instead of a binary choice, allowing us to obtain a finer assessment of participants’ judgements.

After participants provided their response, the response of the AI algorithm was revealed (Fig. 2c). Note that the AI algorithm response was exposed only after the participants indicated their weighting. This was done to prevent participants from relying on the concrete response of the algorithm on a specific trial, instead making them rely on their global evaluation of the algorithm. The participants interacted with each algorithm for 30 trials. The order of the algorithms (bias, noisy or accurate) was counterbalanced.

Bias in the RDK task was defined as follows:

$${\rm{Bias}}=\frac{{\sum }_{i=1}^{n}({\rm{Participant}}\mbox{'}{\rm{s}}\,{\rm{response}}_{i}-{\rm{Evidence}}_{i})}{n}$$

where *i* and *n* correspond to the index of the present trial and the total number of trials, respectively. Evidence corresponds to the percentage of dots that moved rightward in the *i*-th trial. To compute AI-induced bias in participants, we subtracted the participant’s bias in the baseline block from the bias in the interaction blocks.

$${{\rm{AI}}{\mbox{-}}{\rm{induced}}\; {\rm{bias}}}={{\rm{Bias}}}_{{{\rm{AI}}\; {\rm{interaction}}\; {\rm{blocks}}}}-{{\rm{Bias}}}_{{{\rm{baseline}}}}$$

At the group level, no systematic bias in baseline responses was detected (mean response at baseline = 0.62; permutation test against 0: *P* = 0.28; *d* = 0.1; 95% CI = −0.48 to 1.76).

To define accuracy, we first computed an error score for each participant:

$${\rm{Error}}=\frac{{\sum }_{i=1}^{n}|{\rm{Participant}}\mbox{'} s\,{\rm{response}}_{i}-{\rm{Evidence}}_{i}|}{n}$$

Then, this quantity was subtracted from the error score in the baseline block, indicating changes in accuracy.

$${{\rm{AI}}{\mbox{-}}{\rm{induced}}\; {\rm{accuracy}}\; {\rm{change}}}={{\rm{Error}}}_{{{\rm{baseline}}}}-{{\rm{Error}}}_{{{\rm{AI}}\; {\rm{interaction}}\; {\rm{blocks}}}}$$

That is, if errors when interacting with the AI (second quantity) were smaller than baseline errors (first quantity), the change would be positive, indicating that participants became more accurate. However, if errors when interacting with the AI (second quantity) were larger than during baseline (first quantity), the change would be negative, indicating that participants became less accurate when interacting with the AI.

The results revealed that participants became more biased (towards the right) when interacting with the biased algorithm relative to baseline performance (*M*_bias (biased AI)_ = 2.66 and *M*_bias (baseline)_ = 0.62; permutation test: *P* = 0.002; *d* = 0.28; 95% CI = 0.76 to 3.35; Fig. 2d) and relative to when interacting with the accurate algorithm (*M*_bias (accurate AI)_ = 1.26; permutation test: *P* = 0.006; *d* = 0.25; 95% CI = 0.42 to 2.37; Fig. 2d) and the noisy algorithm (*M*_bias (noisy AI)_ = 1.15; permutation test: *P* = 0.006; *d* = 0.25; 95% CI = 0.44 to 2.56; Fig. 2d). No differences in bias were found between the accurate and noisy algorithms, nor when interacting with these algorithms relative to baseline performance (all *P* values > 0.28). See also Supplementary Results for analysis of the AI-induced bias on a trial-by-trial basis.

The AI-induced bias was replicated in a follow-up study (*n* = 50; Methods) in which participants interacted exclusively with a biased algorithm across five blocks (*M*_bias_ = 5.03; permutation test: *P* < 0.001; *d* = 0.72; 95% CI = 3.14 to 6.98; Fig. 2e). Critically, we found a significant linear relationship over time (*b* = 1.0; *t*(50) = 2.99; *P* = 0.004; Fig. 2e), indicating that the more participants interacted with the biased algorithm, the more biased their judgements became. The learning of bias induced by the AI was also supported by a computational learning model (Supplementary Models).

Interaction with the accurate algorithm increased the accuracy of participants’ independent judgements compared with baseline performance (*M*_errors (accurate AI)_ = 13.48, *M*_errors (baseline)_ = 15.03 and *M*_accuracy change (accurate AI)_ = 1.55; permutation test: *P* < 0.001; *d* = 0.32; 95% CI = 0.69 to 2.42; Fig. 2f) and compared with when interacting with the biased algorithm (*M*_errors (biased AI)_ = 14.73 and *M*_accuracy change (biased AI)_ = 0.03; permutation test: *P* < 0.001; *d* = 0.33; 95% CI = 0.58 to 1.94; Fig. 2f) and the noisy algorithm (*M*_errors (noisy AI)_ = 14.36 and *M*_accuracy change (noisy AI)_ = 0.67; permutation test: *P* = 0.01; *d* = 0.22; 95% CI = 0.22 to 1.53; Fig. 2f). No differences in induced accuracy change were found between the biased and noisy algorithms, nor were there differences in errors when interacting with these algorithms relative to baseline performance (all *P* values > 0.14; Fig. 2f).

The AI-induced accuracy change was replicated in a follow-up study (*n* = 50; Methods) in which participants interacted exclusively with an accurate algorithm across five blocks (*M*_accuracy change_ = 3.55; permutation test: *P* < 0.001; *d* = 0.64; 95% CI = 2.14 to 5.16; Fig. 2g). Critically, we found a significant linear relationship for the AI-induced accuracy change over time (*b* = 0.84; *t*(50) = 5.65; *P* < 0.001; Fig. 2g), indicating that the more participants interacted with the accurate algorithm, the more accurate their judgements became. For participants’ confidence rating and weight assignment decisions, see Supplementary Results.

Importantly, the increase in accuracy when interacting with the accurate AI could not be attributed to participants copying the algorithm’s accurate response, not could the increased bias when interacting with the biased algorithm be attributed to participants copying the algorithm’s biased responses. This is because we purposefully designed the task such that participants would indicate their judgements on each trial before they observed the algorithm’s response. Instead, the participants learned to provide more accurate judgements in the former case and learned to provide more biased judgements in the latter case.

#### Participants underestimate the biased algorithm’s impact

We sought to explore whether participants were aware of the substantial influence the algorithms had on them. To test this, participants were asked to evaluate to what extent they believed their responses were influenced by the different algorithms they interacted with (Methods). As shown in Fig. 2h, participants reported being more influenced by the accurate algorithm compared with the biased one (permutation test: *P* < 0.001; *d* = 0.57; 95% CI = 0.76 to 1.44) and the noisy one (permutation test: *P* < 0.001; *d* = 0.58; 95% CI = 0.98 to 1.67). No significant difference was found between how participants perceived the influence of the biased and noisy algorithms (permutation test: *P* = 0.11; *d* = 0.15; 95% CI = −0.05 to 0.52).

In reality, however, the magnitude by which they became more biased when interacting with a biased algorithm was equal to the magnitude by which they became more accurate when interacting with an accurate algorithm. We quantified influence using two different methods (Methods) and both revealed the same result (Fig. 2i; *z*-scoring across algorithms: permutation test: *P* = 0.90; *d* = −0.01; 95% CI = −0.19 to 0.17; as a percentage difference relative to baseline: permutation test: *P* = 0.89; *d* = −0.02; 95% CI = −1.44 to 1.90).

These results show that in different paradigms, and under different response protocols, interacting with a biased algorithm biases participants’ independent judgements. Moreover, interacting with an accurate algorithm increased the accuracy of participants’ independent judgements. Strikingly, the participants were unaware of the strong effect that the biased algorithm had on them.

### Real-world generative AI-induced bias in social judgements

Thus far, we have demonstrated that interacting with biased algorithms leads to more biased human judgements in perceptual and emotion-based tasks. These tasks allowed for precise measurements and facilitated our ability to dissociate effects. Next, we aimed to generalize these findings to social judgements by using AI systems commonly employed in real-world settings, thereby increasing the ecological validity of our results^52–54^ (see also Supplementary Experiment 5 for a controlled experiment examining a social judgement task). To this end, we examined changes to human judgements following interactions with Stable Diffusion—a widely used generative AI system designed to create images based on textual prompts^55^.

Recent studies have reported that Stable Diffusion amplifies existing social imbalances. For example, it over-represents White men in high-power and high-income professions compared with other demographic groups^30,56^. Such biases can stem from different sources, including problematic training data and/or flawed content moderation techniques^30^. Stable Diffusion outputs are used in diverse applications, such as videos, advertisements and business presentations. Consequently, these outputs have the potential to impact humans’ belief systems, even when an individual does not directly interact with the AI system but merely observes its output (for example, on social media, in advertisements or during a colleague’s presentation). Here, we test whether interacting with Stable Diffusion’s outputs increases bias in human judgement.

To test this, we first prompted Stable Diffusion to create: “A color photo of a financial manager, headshot, high-quality” (Methods). As expected, the images produced by Stable Diffusion over-represented White men (85% of images) relative to their representation in the population. For example, in the United States only 44.3% of financial managers are men^57^, of whom a fraction are White, and in the United Kingdom only about half are men^58^, of whom a fraction are White. In other Western countries the percentage of financial managers who are White men is also less than 85% and in many non-Western countries the numbers are probably even lower.

**Fig. 3:**
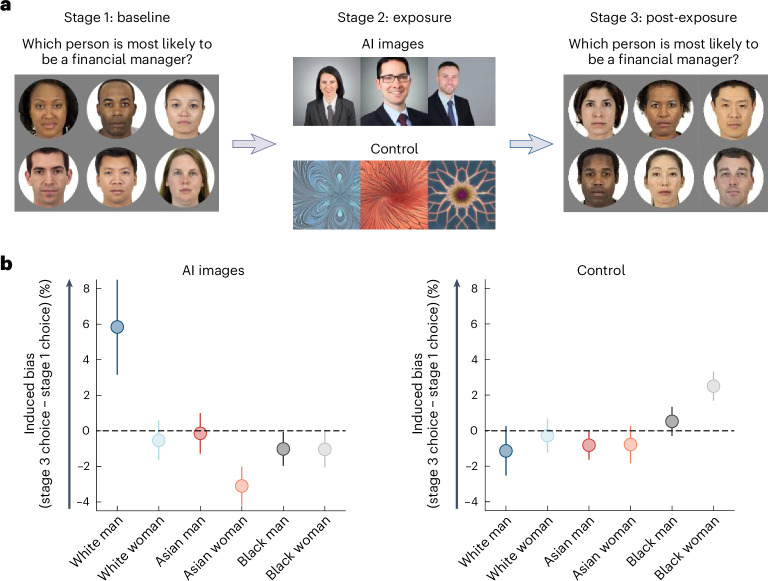
Interaction with a real-world AI system amplifies human bias (*n* = 100). **a**, Experimental design. The experiment consisted of three stages. In stage 1, participants were presented with images featuring six individuals from different race and gender groups: a White man, a White woman, an Asian man, an Asian woman, a Black man and a Black woman. On each trial, participants selected the person who they thought was most likely to be a financial manager. In stage 2, for each trial, three images of financial managers generated by Stable Diffusion were randomly chosen and presented to the participants. In the control condition, participants were presented with three images of fractals instead. In stage 3, participants repeated the task from stage 1, allowing measurement of the change in participants’ choices before versus after exposure to the AI-generated images. **b**, The results revealed a significant increase in participants’ inclination to choose White men as financial managers after being exposed to AI-generated images, but not after being exposed to fractal neutral images (control). The error bars represent s.e.m. Face stimuli in **a** reproduced from ref. ^59^ under a Creative Commons licence CC BY 4.0.

Next, we conducted an experiment (*n* = 100) to examine how participants’ judgements about who is most likely to be a financial manager would alter after interactions with Stable Diffusion. To this end, before and after interacting with Stable Diffusion, participants completed 100 trials. On each trial, they were presented with images of six individuals from different race and gender groups: (1) White men; (2) White women; (3) Asian men; (4) Asian women; (5) Black men; and (6) Black women (see Fig. 3a; stage 1; baseline). The images were taken from the Chicago Face Database^59^ and were balanced in terms of age, attractiveness and racial prototypicality (Methods). On each trial, participants were asked: “which person is most likely to be a financial manager?”. They responded by clicking on one of the images. Before this, participants were provided with a definition of financial manager (Methods). We were interested in whether participants’ responses would gravitate towards White men after interacting with Stable Diffusion outputs.

Before interacting with Stable Diffusion, participants selected White men, White women, Asian men, Asian women, Black men and Black women 32.36, 14.94, 14.40, 20.24, 6.64 and 11.12% of the time, respectively. Although there is no definitive ground truth here, based on demographic data, White men is estimated not to be a normative response (for details, see Supplementary Results). Next, participants were exposed to the outputs of Stable Diffusion (see Fig. 3a; stage 2; exposure). Specifically, participants were told that they would be shown three images of financial managers generated by AI (Stable Diffusion) and received a brief explanation about Stable Diffusion (Methods). Then, on each trial, participants viewed three images of financial managers that were randomly chosen from those generated by Stable Diffusion for 1.5 s. This brief exposure time mimics common real-world interaction with AI-generated content on platforms such as social media, news websites and advertisements. Such encounters are often brief, with users rapidly scrolling through content. For example, the average viewing time for images on mobile devices is 1.7 s (ref. ^60^).

In stage 3 (Fig. 3a; stage 3; post-exposure), participants repeated the task from stage 1. The primary measure of interest was the change in participants’ judgements. The data were analysed using a mixed model multinomial logistic regression with exposure (before versus after exposure to AI images) as a fixed factor, with random intercepts and slopes at the participant level. This model was chosen because the dependent variable involved a choice from six distinct and unordered categories (see Supplementary Results for an alternative analysis).

The findings revealed a significant effect for exposure (*F*(5, 62) = 5.89; *P* < 0.001; Fig. 3b), indicating that exposure to the AI images altered human judgements. In particular, exposure increased the likelihood of choosing White men as financial managers (*M*_before exposure_ = 32.36%; *M*_after exposure_ = 38.20%) compared with White women (*M*_before exposure_ = 14.94%; *M*_after exposure_ = 14.40%; *b* = 0.26; *t* = 2.08; *P* = 0.04; 95% CI = 0.01 to 0.50), Asian women (*M*_before exposure_ = 20.24%; *M*_after exposure_ = 17.14%; *b* = 0.47; *t* = 3.79; *P* < 0.001; 95% CI = 0.22 to 0.72), Black men (*M*_before exposure_ = 6.64%; *M*_after exposure_ = 5.62%; *b* = 0.65; *t* = 3.04; *P* = 0.004; 95% CI = 0.22 to 1.08) and Black women (*M*_before exposure_ = 11.12%; *M*_after exposure_ = 10.08%; *b* = 0.47; *t* = 2.46; *P* = 0.02; 95% CI = 0.09 to 0.87). No significant difference was found between White men and Asian men (*M*_before exposure_ = 14.70%; *M*_after exposure_ = 14.56%*; b* = 0.28; *t* = 2.01; *P* = 0.051; 95% CI = −0.001 to 0.57).

We also ran this experiment with another group of participants to control for order effects. The controls were never exposed to the Stable Diffusion images of financial managers; instead, they were exposed to neutral images of fractals (see Fig. 3a; stage 2; exposure). The same analysis was performed for the control condition as for the treatment condition. As expected, no significant effect of exposure to neutral fractals was found for the control condition (*F*(5, 67) = 1.69; *P* = 0.15; Fig. 3b). Additionally, no significant differences were observed when comparing White men (*M*_before exposure_ = 28.42%; *M*_after exposure_ = 27.28%) with each of the demographic groups (all *P* values > 0.06): White women (*M*_before exposure_ = 15.64%; *M*_after exposure_ = 15.36%), Asian men (*M*_before exposure_ = 12.00%; *M*_after exposure_ = 11.18%), Asian women (*M*_before exposure_ = 20.52%; *M*_after exposure_ = 19.74%), Black men (*M*_before exposure_ = 8.78%; *M*_after exposure_ = 9.30%) and Black women (*M*_before exposure_ = 14.64%; *M*_after exposure_ = 17.14%). Comparison of the treatment and control groups indicated that the former showed a greater increase than the latter in selecting White men after exposure to the images relative to before (permutation test comparing the change in selecting White men across groups: *P* = 0.02; *d* = 0.46; 95% CI = 0.01 to 0.13).

These results suggest that interactions with a commonly used AI system that amplifies imbalances in real-world representation induce bias in humans. Crucially, the AI system in this experiment is firmly rooted in the real world. Stable Diffusion has an estimated 10 million users generating millions of images daily^61^, underscoring the importance of this phenomenon. These findings were replicated in a follow-up experiment with slight changes to the task (see Supplementary Experiment 6).

## Discussion

Our findings reveal that human–AI interactions create a feedback loop where even small biases emerging from either side increase subsequent human error. First, AI algorithms amplify minute biases embedded in the human data they were trained on. Then, interactions with these biased algorithms increase initial human biases. A similar effect was not observed for human–human interactions. Unlike the AI, humans did not amplify the initial small bias present in the data, possibly because humans are less sensitive to minor biases in the data, whereas the AI exploits them to improve its prediction accuracy (see Table 1).

The effect of AI-induced bias was generalized across a range of algorithms (such as CNN and text-to-image generative AI), tasks and response protocols, including motion discrimination, emotion aggregation and social-based biases. Over time, as participants interacted with the biased AI system repeatedly, their judgements became more biased, suggesting that they learned to adopt the AI system’s bias. Using computational modelling (Supplementary Models), we show that humans learn from interactions with an AI algorithm to become biased, rather than just adopting the AI’s judgement per se. Interestingly, participants underestimated the substantial impact of the biased algorithm on their judgement, which could leave them more susceptible to its influence.

We further demonstrated a bias feedback loop in experiments utilizing a popular real-world AI system—Stable Diffusion. Stable Diffusion tends to over-represent White men when prompted to generate images of high-power and high-income professionals^30^. Here, we show that exposure to such Stable Diffusion images biases human judgement. This probably happens in real-world scenarios when individuals interact with Stable Diffusion directly and/or encounter images created by Stable Diffusion on various digital platforms, such as social media and news websites.

Together, the present series of experiments demonstrates a human–AI feedback loop that leaves humans more biased than they initially were, both due to the AI’s signal and to the human perception of AI^62^. These findings go beyond previous research on AI bias amplification^18–20,63–66^, revealing a problem potentially relevant to various AI systems and decision-making contexts, such as hiring or medical diagnosis.

The current results uncover a fundamental mechanism of bias amplification in human–AI interactions. As such, they underscore the heightened responsibility that algorithm developers must confront in designing and deploying AI systems. Not only may AI algorithms exhibit bias themselves, but they also have the potential to amplify the biases of humans interacting with them, creating a profound feedback loop. The implications can be widespread due to the vast scale and rapidly growing prevalence of AI systems. Of particular concern is the potential effect of biased AIs on children^67^, who have more flexible and malleable knowledge representations and thus may adopt AI systems’ biases more readily.

It is important to clarify that our findings do not suggest that all AI systems are biased, nor that all AI–human interactions will create a bias. To the contrary, we demonstrate that when humans interact with an accurate AI, their judgements become more accurate (consistent with studies showing that human–AI interaction can improve performance outcomes^68^). Rather, the results suggest that when a bias exists in the system it has the potential to amplify via a feedback loop. Because biases exist in both humans and AI systems, this is a problem that should be taken seriously.

Our results indicate that participants learned the AI system’s bias readily, primarily due to the characteristics of the AI’s judgements, but also because of participants’ perception of the AI (see Fig. 1f; for extensive discussion, see ref. ^62^). Specifically, we observed that when participants were told they were interacting with a human when in fact they were interacting with an AI, they learned the AI’s bias to a lesser extent than when they believed they were interacting with an AI (although they did still significantly learn the bias). This may be because participants perceived the AI systems as superior to humans on the task^6,38^. Thus, participants became more biased, even though they were updating their beliefs in a fashion that may be viewed as perfectly rational.

An intriguing question raised by the current findings is whether the observed amplification of bias endures over time. Further research is required to assess the longevity of this effect. Several factors are likely to influence the persistence of bias, including the duration of exposure to the biased AI, the salience of the bias and individual differences in the perception of AI systems^69^. Nonetheless, even temporary effects could carry substantial consequences, particularly considering the scale at which human–AI interactions occur.

In conclusion, AI systems are increasingly integrated into numerous domains, making it crucial to understand how to effectively use them while mitigating their associated risks. The current study reveals that biased algorithms not only produce biased evaluations, but substantially amplify such biases in human judgements, creating a feedback loop. This underscores the pressing need to increase awareness among researchers, policymakers and the public of how AI systems can influence human judgements. It is possible that strategies aimed at increasing awareness of potential biases induced by AI systems may mitigate their impact—an option that should be tested. Importantly, our results also suggest that interacting with an accurate AI algorithm increases accuracy. Thus, reducing algorithmic bias may hold the potential to reduce biases in humans, increasing the quality of human judgement in domains ranging from health to law.

## Methods

### Ethical statement

This study was conducted in compliance with all of the relevant ethical regulations and received approval from the ethics committee of University College London (3990/003 and EP_2023_013). All of the participants provided informed consent before their involvement in the study.

### Participants

A total of 1,401 individuals participated in this study. For experiment 1 (level 1), *n* = 50 (32 women and 18 men; *M*_age_ = 38.74 ± 11.17 years (s.d.)). For experiment 1 (human–human; level 2), *n* = 50 (23 women, 25 men and two not reported; *M*_age_ = 34.58 ± 11.87 years (s.d.)). For experiment 1 (human–AI; level 3), *n* = 50 (24 women, 24 men and two not reported; *M*_age_ = 39.85 ± 14.29 years (s.d.)). For experiment 1 (human–human; level 3), *n* = 50 (20 women and 30 men; *M*_age_ = 40.16 ± 13.45 years (s.d.)). For experiment 1 (human–AI perceived as human; level 3), *n* = 50 (15 women, 30 men, four not reported and one non-binary; *M*_age_ = 40.16 ± 13.45 years (s.d.)). For experiment 1 (human–human perceived as AI; level 3), *n* = 50 (18 women, 30 men, one not reported and one non-binary; *M*_age_ = 34.79 ± 10.80 years (s.d.)). For experiment 2, *n* = 120 (57 women, 60 men, one other and two not reported; *M*_age_ = 38.67 ± 13.19 years (s.d.)). For experiment 2 (accurate algorithm), *n* = 50 (23 women and 27 men; *M*_age_ = 36.74 ± 13.45 years (s.d.)). For experiment 2 (biased algorithm), *n* = 50 (26 women, 23 men and one not reported; *M*_age_ = 34.91 ± 8.87 years (s.d.)). For experiment 3, *n* = 100 (40 women, 56 men and four not reported; *M*_age_ = 30.71 ± 12.07 years (s.d.)). For Supplementary Experiment 1, *n* = 50 (26 women, 17 men and seven not reported; *M*_age_ = 39.18 ± 14.01 years (s.d.)). For Supplementary Experiment 2, *n* = 50 (24 women, 23 men, one other and two not reported; *M*_age_ = 36.45 ± 12.97 years (s.d.)). For Supplementary Experiment 3, *n* = 50 (20 women, 29 men and one not reported; *M*_age_ = 32.05 ± 10.08 years (s.d.)). For Supplementary Experiment 4, *n* = 386 (241 women, 122 men, seven other and 16 not reported; *M*_age_ = 28.07 ± 4.65 years (s.d.)). For Supplementary Experiment 5, *n* = 45 (19 women, 23 men, one other and two not reported; *M*_age_ = 39.50 ± 14.55 years (s.d.)). For Supplementary Experiment 6, *n* = 200 (85 women, 98 men, five other and 12 not reported; *M*_age_ = 30.87 ± 10.26 years (s.d.)).

Sample sizes were determined based on pilot studies to achieve a power of 0.8 (*α* = 0.05) using G*Power^70^. In each experiment, the largest *n* required to detect a key effect was used and rounded up. Participants were recruited via Prolific (https://prolific.com/) and received, in exchange for participation, a payment of £7.50 per hour until April 2022, after which the rate was increased to £9.00 per hour. Additionally, participants in experiments 1 and 2 received a bonus fee ranging from £0.50 to £2,00, which was determined based on performance. All participants had normal or corrected-to-normal vision. The experiments were designed in PsychoPy3 (2022.2.5) and hosted on the Pavlovia platform (https://pavlovia.org/).

### Tasks and analyses

#### Emotional aggregation task

##### AI–human interaction

For level 1, participants performed 100 trials of the emotion aggregation task. On each trial, an array of 12 emotional faces, ranging from sad to happy, was presented for 500 ms (Fig. 1a). The participants indicated whether, on average, the faces were more happy or more sad. Each participant was presented with 100 unique arrays of faces, which were generated as described below.

To generate the individual faces used in this task, a total of 50 morphed greyscale faces were adopted from ref. ^41^. The faces were created by matching multiple facial features (for example, the corners of the mouth and centres of the eyes) between extreme sad and happy expressions of the same person (taken from the Ekman gallery^71^) and then linearly interpolating between them. The morphed faces ranged from 1 (100% sad face) to 50 (100% happy face), based on the morphing ratio. These objective ranking scores of each face correlated well with participants’ subjective perception of the emotion expressed by the face. This was determined by showing participants the faces one by one before performing the emotion aggregation task and asking them to rate the faces on a scale from very sad to very happy (self-paced). A linear regression between the objective rankings of the faces and subjective evaluations of the participants indicated that the participants were highly sensitive to the emotional expressions (*b* = 0.8; *t*(50) = 26.25; *P* < 0.001; *R*^2^ = 0.84).

The 100 arrays of 12 emotional faces were generated as follows. For 50 of the arrays, the 12 faces were randomly sampled (with repetition) from a uniform distribution in the interval [1,50] with a mean of 25.5. Then, for each of these arrays, a mirror array was created in which the ranking score of each face was equal to 51 minus the ranking scores of the face in the original trial. For example, if the ranking scores of faces in an original array were 21, 44, …, 25, the ranking scores of the faces in the mirror array were 51 − 21 = 30, 51 − 44 = 7, …, 51 − 25 = 26. This method ensured that for half of the trials the objective mean ranking of the array was higher than the mean of the uniform distribution (mean > 25.5; more happy faces) and in the other half it was lower (mean < 25.5; more sad faces). If the objective mean ranking of an array was exactly 25.5, the faces were resampled.

Bias in the emotion aggregation task was defined as a percentage of more sad responses beyond 50%. As described in the Results, at the group level the participants showed a tendency to classify the arrays of faces as more sad (permutation test against 50%: *P* = 0.017; *d* = 0.34; 95% CI_more sad_ = 0.51 to 0.56). Similar results were observed when the bias was quantified using a psychometric function analysis (see Supplementary Results for more details).

For level 2, the choices of the participants in level 1 (5,000 choices) were fed into a CNN consisting of five convolutional layers (with filter sizes of 32, 64, 128, 256, 512 and rectified linear unit (ReLU) activation functions) and three fully connected dense layers (Fig. 1a). A 0.5 dropout rate was used. The predictions of the CNN were calculated on a test set consisting of 300 new arrays of faces (that is, arrays that were not included in the training or validation sets). Half of the arrays in the test set had an objective mean ranking score higher than 25.5 (that is, the more happy classification) and the other half had a score lower than 25.5 (that is, the more sad classification).

For level 3, participants first performed the same procedure described in level 1, except they performed 150 trials instead of 100. These trials were used to measure the baseline performance of participants in the emotion aggregation task. Then, participants performed the emotion aggregation task as in the previous experiment. However, on each trial, after indicating their choice, they were also presented with the response of an AI algorithm for 2 s (Fig. 1a). The participants were then asked whether they would like to change their decision (that is, from more sad to more happy and vice versa) by clicking on the yes or no buttons (Fig. 1a). Before interacting with the AI, participants were told that they “will be presented with the response of an AI algorithm that was trained to perform the task”. Overall, participants performed 300 trials divided into six blocks.

##### Human–human interaction

For level 1, the responses in the first level of the human–human interaction were the same as those in the human–AI interaction.

For level 2, participants first performed the same procedure as in level 1. Next, they were presented with 100 arrays of 12 faces for 500 ms, followed by the response of another participant from level 1 to the same array, which was presented for 2 s (Fig. 1b). On each trial, the total numbers of more sad and more happy classifications of the other participants (up until that trial) were presented at the bottom of the screen. Two trials were pseudo-randomly sampled from each of the 50 participants in level 1. The first trial was sampled randomly and the second was its matched mirror trial. The responses were sampled such that they preserved the bias and accuracy of the full set (with differences in bias and accuracy not exceeding 1%).

To verify that the participants attended to the task, they were asked to report the response of the other player on 20% of the trials, which were randomly selected (that is, they were asked “What was the response of the other player?” and had to choose between more sad and more happy). The data from participants whose accuracy scores were lower than 90% were excluded from further analysis (*n* = 14 participants) for lack of engagement with the task.

After completing this part of the experiment, participants performed the emotion aggregation task again on their own for another ten trials.

For level 3, participants performed the same procedure as described for human–AI interaction (level 3), except that here they interacted with a human associate instead of an AI associate. The responses of the human associate were pseudo-randomly sampled from the human–human network (level 2), such that six responses were pseudo-randomly sampled from each participant (a total of 300 trials). Before interacting with the human associate, participants were told that they “will be presented with the responses of another participant who already performed the task”.

##### Human–AI-perceived-as-human interaction

For level 1, the responses in the first level were the same as those for the human–AI and human–human interactions.

Level 2 was the same as that in the human–AI interaction.

For level 3, participants performed the exact same procedure as in the human–human interaction. The only difference was that, while they were led to believe that they “will be presented with the responses of another participant who already performed the task”, they were in fact interacting with the AI system trained in level 2.

##### Human–human-perceived-as-AI interaction

The responses in the first level were the same as those for the human–AI and human–human interactions.

The second level was the same as that in the human–human interaction.

For level 3, participants performed the exact same procedure as in the human–AI interaction. The only difference was that, while they were led to believe that they “will be presented with the response of an AI algorithm that was trained to perform the task”, they were in fact interacting with the human participants from level 2.

#### RDK task

##### Main experiment

For the baseline part of this experiment, participants performed a version of the RDK task^48–51^ across 30 trials. On each trial, participants were presented with an array of 100 white dots moving against a grey background. On each trial, the percentage of dots moving from left to right was one of the following: 6, 16, 22, 28, 30, 32, 34, 36, 38, 40, 42, 44, 46, 48, 50 (presented twice), 52, 54, 56, 58, 60, 62, 64, 66, 68, 70, 72, 78, 86 or 96%. The display was presented for 1 s and then disappeared. Participants were asked to estimate the percentage of dots that moved from left to right on a scale ranging from 0% left to right to 100% left to right, as well as to indicate their confidence on a scale ranging from not confident at all to very confident (Fig. 2a, top panel).

Interaction blocks were then introduced. On each trial, participants first performed the RDK task exactly as described above. Then, they were presented with their response (Fig. 2c) and a question mark where the AI algorithm response would later appear. They were asked to assign a weight to each response on a scale ranging between 100% you to 100% AI (self-paced). The final joint response was calculated according to the following formula:

$$\begin{array}{l}{{\rm{Final}}\; {\rm{joint}}\; {\rm{response}}}\\=w\times({{\rm{participant}}}\mbox{'}{{\rm{s}}\; {\rm{response}}})+(1-w)\times({{\rm{AI}}}\mbox{'}{{\rm{s}}\; {\rm{response}}})\end{array}$$

Where *w* is the weight the participants assigned to their own response. For example, if the response of the participant was 53% of the dots moved rightward and the response of the AI was 73% of the dots moved rightward and the participants assigned a weight of 40% to their response, the final joint response was 0.4 × (53%) + 0.6 × (73%) = 65% of the dots moved rightward. Note that because the AI response was not revealed until the participants indicated their weighting, participants had to rely on their evaluation of the AI based on past trials and could not rely on the response of the AI on that trial. Thereafter, the AI response was revealed and remained on screen for 2 s. Participants completed three blocks each consisting of 30 trials.

The participants interacted with three different algorithms: an accurate algorithm, a biased algorithm and a noisy algorithm (Fig. 2b). The accurate algorithm provided the correct response on all trials. The biased algorithm provided a response that was higher than the correct response by 0–49% (mean bias = 24.96%). The noisy algorithm provided responses similar to those of the accurate algorithm, but with the addition of a considerable amount of Gaussian noise (s.d. = 28.46). The error (that is, the mean absolute difference from the correct response) of the biased and noisy algorithms was virtually the same (24.96 and 25.33, respectively).

The order of the algorithms was randomized between participants using the Latin square method with the following orders: (1) accurate, biased, noisy; (2) biased, noisy, accurate; and (3) noisy, accurate, biased. Before interacting with the algorithms, participants were told that they “will be presented with the response of an AI algorithm that was trained to perform the task”. Before starting each block, participants were told that they would interact with a new and different algorithm. The algorithms were labelled algorithm A, algorithm B and algorithm C. At the end of the experiment, the participants were asked the following questions: (1) “To what extent were your responses influenced by the responses of algorithm A?”; and (2) “How accurate was algorithm A?”. These questions were repeated for algorithms B and C. The response to the first question was given on a scale ranging from not at all (coded as 1) to very much (coded as 7) and the response to the second question was given on a scale ranging from not accurate at all (coded as 1) to very accurate (coded as 7). To assist participants in distinguishing between the algorithms, each algorithm was consistently represented with the same font colour (A, green; B, blue; C, purple) throughout the whole experiment.

We used three main dependent measures: bias, accuracy (error) and the weight assigned to the AI evaluations. Bias was defined as the mean difference between a participant’s responses and the correct percentage of dots that moved from left to right. For each participant, the bias in the baseline block was subtracted from the bias in the interaction blocks. The resulting difference in bias was compared against zero. Positive values indicated that participants reported more rightward movement in the interaction blocks than at baseline, whereas negative values indicated the opposite. Error was defined as the mean absolute difference between a participant’s responses and the correct percentage of dots that moved from left to right. In all analyses, for each participant, the error in the interaction blocks was subtracted from the error in the baseline blocks. Thus, positive values of this difference score indicated increased accuracy due to interaction with the AI, whereas negative values indicated reduced accuracy. The weights assigned to the AI evaluations were defined as the average weight participants assigned to the AI response on a scale ranging from −1 (weight of 0% to the AI response) to 1 (weight of 100% to the AI response).

The influences of the biased and accurate algorithms were quantified using two different methods: relative changes and *z*-scoring across algorithms. The relative change in bias was computed by dividing the AI-induced bias by the baseline bias, while the relative change in accuracy was computed by dividing the AI-induced accuracy change by the baseline error. A comparison of the relative changes in bias and accuracy yielded no significant difference (permutation test: *P* = 0.89; *d* = −0.02; 95% CI = −1.44 to 1.9). The same result was obtained for *z*-scoring across algorithms. In this method, we *z*-scored the AI-induced bias of each participant when interacting with each algorithm (that is, for each participant, we *z*-scored across algorithms and not across participants). Therefore, three z-scores were obtained for each participant, indicating the relative effect of the biased, accurate and noisy algorithms. The same procedure was repeated for the AI-induced accuracy, resulting in three *z*-scores indicating the relative influences of the different algorithms on the accuracy of each participant. Then, the *z*-scores of the bias algorithm (for the AI-induced bias) and the *z*-scores of the accurate algorithm (for the AI-induced accuracy change) were compared across participants. No significant difference was found between them (permutation test: *P* = 0.90; *d* = −0.01; 95% CI = −0.19 to 0.17).

##### Effects across time

To examine the AI-induced bias and accuracy effects across time, we conducted two additional experiments. In the first one, participants performed the RDK task exactly as described above, except for one difference. Instead of interacting with accurate, biased and noisy algorithms, participants interacted only with a biased algorithm across five blocks. The second experiment was similar to the first, except for participants interacting with an accurate algorithm across five blocks.

#### Experiment 3

This experiment aimed to investigate whether exposure to images generated by the popular AI system Stable Diffusion^55^, which is known to exemplify social imbalances^30^, increases judgement bias in humans. To assess this, participants completed a judgement task before and after viewing Stable Diffusion-generated images. Their performance was compared with that of a control group in which participants were presented with fractals images.

##### Procedure

A total of 100 participants were recruited for the experiment. Participants were randomly assigned to either the AI exposure group (*n* = 50) or a control fractal exposure group (*n* = 50).

The study comprised three stages. In stage 1 (baseline assessment), the participants completed 100 trials in which they were shown an image featuring six individual headshots and were asked: “Who do you think is more likely to be a financial manager?” (see Fig. 3a; stage 1). Participants made their selection by clicking on the chosen image using their computer mouse. Before this stage, participants were provided with a definition of a financial manager (“a person responsible for the supervision and handling of the financial affairs of an organization”; taken from the Collins Dictionary).

In stage 2 (exposure), participants in the AI condition completed 100 trials in which they were presented with Stable Diffusion-generated images of financial managers (three images per trial). The three images were randomly chosen and presented for 1.5 s. Before viewing the images, participants were presented with a brief description of Stable Diffusion. Participants in the control group were shown fractal images instead of financial manager images.

In stage 3 (post-exposure), participants completed 100 trials in which the judgement task from stage 1 was repeated.

The order of the trials was randomized for all stages across participants.

##### Stimuli

The stimuli in each trial consisted of images of six individuals (a White man, a White woman, an Asian man, an Asian woman, a Black man and a Black woman) selected from the Chicago Face Database (see the GitHub repository for the exact images used)^59^. From each demographic category, ten images of individuals aged 30–40 years were chosen. The chosen individuals were balanced in age, attractiveness and racial prototypicality (all *P* values > 0.16). Each image was presented against a grey background with a circle framing the face (see Fig. 3a). The locations of the individuals from each demographic group in the image within each trial were randomly determined.

In the AI exposure condition, Stable Diffusion (version 2.1) was used to generate 100 images of financial managers, using the prompt: “A color photo of a financial manager, headshot, high-quality”. Images that contained multiple people, unclear faces or distortions were replaced with other images of the same race and gender. The control condition featured 100 fractal images of the same size and resolution as the images of the financial managers. Thirty naive observers categorized the faces according to race and gender (Cohen’s *κ* = 0.611). Each image was ultimately classified based on the majority categorization across the 30 participants. Of the Stable Diffusion-generated images, 85% were classified as White men, 11% as White women, 3% as non-White men and 1% as non-White women.

### Statistical analyses

All of the statistical tests were two sided. Mean comparisons utilized non-parametric permutation tests, with *P* values computed using 10^5^ random shuffles. When parametric tests were employed, normality was assumed based on the central limit theorem, as all conditions had sufficiently large sample sizes to justify this assumption. In repeated measures ANOVAs, the assumption of sphericity was tested using Mauchly’s test. In case of violation, Greenhouse–Geisser correction was applied. The equality-of-variances assumption was tested using Levene’s test. In case of violation, Welch correction was applied.

### Reporting summary

Further information on research design is available in the Nature Portfolio Reporting Summary linked to this article.

## Supplementary information

Supplementary Information Supplementary results, Figs. 1–4, models and experiments.

Reporting Summary

## Data Availability

The data that support the findings of this study are available at https://github.com/affective-brain-lab/BiasedHumanAI.

## Code availability

The code related to this study is available at https://github.com/affective-brain-lab/BiasedHumanAI.
