# Supplementary material for: How human–AI feedback loops alter human perceptual, emotional and social judgements
Source: Nat Hum Behav. 2024 Dec 18;9(2):345–59. doi: 10.1038/s41562-024-02077-2 (PMC11860214; doi:10.1038/s41562-024-02077-2)
Supplement: Supplementary file 1 — Supplementary results, Figs. 1–4, models and experiments. [file 41562_2024_2077_MOESM1_ESM.pdf]

nature human behaviour

Supplementary information

https://doi.org/10.1038/s41562-024-02077-2

How human–AI feedback loops alter human perceptual, emotional and social judgements

In the format provided by the authors and unedited

Supplementary Results

Participants showed high sensitivity to the emotion expressed by the faces

Before performing the emotion aggregation task, participants in Level 1 were presented with 50 morphed faces (adopted from ref. [1]). The morphed faces were presented one-by-one and ranged from 1 (100% sad face) to 50 (100% happy face). The participants were asked to rate the faces on a scale ranging from 'Very sad' to 'Very happy' (converted to 1 to 50 scale). To examine the sensitivity of the participants to the emotions expressed by the single faces, we conducted a mixed-effects linear regression, predicting the subjective evaluations of the participants from the 'objective' rankings of the morphed faces (i.e., their ranking from 1 to 50, see **Methods/Individual faces**) as a fixed factor and random intercepts and slopes at the participant level. The regressions revealed that the participants were highly sensitive to the emotional expressions of the faces (b = 0.8, $t(50)$ = 26.25, p < 0.001, 95% CI [0.75, 0.85]).

Estimation of the 'more sad' classification bias using psychometric function analysis

In the main text, bias in the emotion aggregation task was defined by comparing the percentage of 'more sad' responses to chance (50%). In this section, we show that the results are robust to a different bias measure, based on a psychometric function analysis. To this end, we performed mixed-model logistic regressions predicting the classifications of the participants (coded as 0 – 'more happy' and 1 – 'more sad') from the mean objective ranking score of each array of 12 faces as a fixed factor and random intercepts and slopes at the participant level. The regressions were conducted separately for the data of the human participants in Levels 1, 2 (Human-Human interaction) and 3 (Human-AI interaction, Human-Human interaction, Human-AI-perceived-as-human interaction and Human-Human-perceived-as-AI interaction), as well as for the predictions of the AI in Level 2 (Human-AI interaction).

For each regression model we extracted the indifference point (i.e., the point at which the probability of classifying an array as 'more sad' or 'more happy' is equal to 50%). The mean objective ranking score of each array (scale of 1-50) was converted to a normalized scale ranging from -1 to 1 using the following formula:

$$Normalized\ Mean\ emotion_i = \frac{25.5 - Mean\ emotion_i}{24.5}$$

Thus, an unbiased agent (human or AI) would have an indifference point of 0, whereas a biased agent would have an indifference point higher than 0 (a bias toward 'more sad' responses) or lower than 0 (a bias toward 'more happy' responses). The confidence intervals (95%) of the indifference points were calculated using bootstrapping method (1,000 bootstraps).

The psychometric function analysis yielded the same results as the ones reported in the main text. The mean indifference point of the human participants in Level 1 was higher than 0 (*M* = 0.032, 95% CI [0.018, 0.047]), indicating a bias toward 'more sad' responses. The mean indifference point of the AI in Level 2 was also higher than 0, and critically higher than that of the participants in Level 1 (*M* = 0.11, 95% CI [0.073, 0.16]), indicating that the AI amplified the human participants bias. Finally, the mean indifference point of the participants in Level 3 of the Human-AI condition (*M* = 0.075, 95% CI [0.065, 0.084]), was higher than that of the participants in Level 3 of the Human-AI-perceived-as-human condition (*M* = 0.054, 95% CI [0.043, 0.063]), the Human-Human-perceived-as-AI condition (*M* = 0.020, 95% CI [0.009, 0.031]), as well as of the indifference point of participants in the Human-Human condition (*M* = 0.016, 95% CI [0.007, 0.024]).

Convolutional neural network models

In this section, we show that AI bias amplification takes place across different architectures of the convolutional neural networks, including the commonly used ResNet network[2]. The models were trained on the 5,000 arrays that were presented to the participants in Level 1 (5,000 arrays = 50 participants × 100 arrays), with class labels defined based on the human classifications. The models were evaluated using 10 out-of-sample test sets with statistical properties similar to the one used in the main text.

We begin by examining the model described in the main text. As a reminder, this model consisted of five convolution layers with filter sizes of 32, 64, 128, 256 and 512. ReLU activation was used for the convolution layers. The fully connected block consisted of dense layers with 1024, 512 and 128 neurons, along with a softmax activation layer. A 0.5 dropout rate was used. This model showed a mean bias of 11.6% ± 9.0% *SD*.

We explored three additional model architectures. The first included six convolution layers with 32, 64, 128, 256, 512 and 1024 filters. ReLU activation was used for the convolution layers, and the max pooling elements had a size of 2×2. The fully connected block contained two dense layers with 512, 128, along with a softmax activation layer. A 0.5 dropout rate was used. This model showed a mean bias of 9.7% ± 6.7% *SD*.

The second model mirrored the previous one, but the dropout layers were removed. This model showed a mean bias of 13.4% ± 7.1% *SD*.

The third model was based on the ResNet50 architecture[2] to which we added three fully connected layers. This model showed a mean bias of 9.8% ± 10.7% *SD*.

Analysis of the induced bias and accuracy change while controlling for time

The analysis of the data of Exp. 2 did not account for learning effects within the blocks. Therefore, we conducted an additional control analysis of the data of Exp. 2, while controlling for time. To this end, we used two separate mixed model linear regressions.

First, we examine if bias alters over the block by conducting a linear mixed model predicting bias on a trial by-trial-basis (defined the difference between the response of the participants and the actual number of dots moving rightward within each trial) from: (i) the type of agent the participant interacted with: biased, noisy and accurate. To represent this categorical variable in the model, two dummy-coded variables were created: one dummy variable compared the accurate agent to the biased one, while the other dummy variable compared the noisy agent to the biased agent. (ii) the evidence (the actual number of dots moving from left to right) and (iii) trial number within each condition (the sequential trial number within each block, ranging from 1 to 30). All were included as fixed factors with random intercepts and slopes. The regression analysis replicated the results of Exp. 2 reported in the main text, showing that participants were more biased when interacting with the bias agent than when interacting with the accurate agent ($b_{Accurate\ vs.\ Biased} = -1.38$, $t(143) = -3.00$, $p = 0.003$, 95% CI [-2.30, -0.47]) and the noisy agent ($b_{Noisy\ vs.\ Biased} = -1.50$, $t(143) = -2.99$, $p = 0.003$, 95% CI [-2.49, -0.51]). Additionally, a significant effect was found for evidence ($b_{Evidence} = -0.33$, $t(120) = -13.22$, $p < 0.001$, 95% CI [-0.38, -0.28]) and for time ($b_{Time} = 0.04$, $t(120) = 2.42$, $p = 0.017$, 95% CI [0.008, 0.08]).

Second, we conducted the same analysis as above, but this time predicting error (defined the absolute difference between the response of the participants and the actual number of dots moving rightward within each trial). In this analysis, the type of agent was dummy coded using the accurate agent as the reference category (i.e., one dummy variable compared the biased agent to the accurate agent, while the other one compared the noisy agent to the accurate agent). Again, the analysis replicated the results of Exp. 2 showing that when interacting with the accurate agent, participants had lower error rates than when interacting with the biased agent ($b_{Biased\ vs.\ Accurate} = 1.25$, $t(153) = 3.75$, $p < 0.001$, 95% CI [0.59, 1.90]) and the noisy agent ($b_{Noisy\ vs.\ Accurate} = 0.87$, $t(153) = 2.79$, $p = 0.007$, 95% CI [0.24, 1.50]). In addition, there was a significant effect for evidence ($b_{Evidence} = -0.05$, $t(120) = -3.71$, $p < 0.001$, 95% CI [-0.08, -0.02]) and for time ($b_{Time} = -0.04$, $t(120) = -3.79$, $p < 0.001$, 95% CI [-0.07, -0.02]).

Confidence ratings and assigned weights to the AI's evaluations in Exp. 2

In addition to examining differences in induced bias and accuracy changes in Exp. 2, we also analyzed differences in participants' confidence and the weights they assigned to the AI's evaluations, while interacting with the accurate, biased and noisy algorithms. Assigned weights were defined as the weight participants gave to the AI evaluations on a scale ranging from -1 ('100% You', i.e., a weight of 0% was assigned to the AI response) to 1 ('100% AI', i.e., a weight of 100% was assigned to the AI response).

Participants assigned higher weights to the AI response when interacting with the accurate algorithm, as compared to the biased algorithm ($M\_Assigned\ Weights_{\text{accurate AI}} = 0.09$, $M\_Assigned\ Weights_{\text{biased AI}} = -0.09$, $P\ permutation < 0.001$, $d = 0.40$, 95% CI [0.10, 0.26])

and the noisy algorithm (*M_ Assigned Weights* $_{\text{noisy AI}}$ = 0.09, *P permutation* < 0.001, *d* = 0.43, 95% CI [0.10, 0.25]). No differences in assigned weights were found between the biased and noisy algorithms (*P permutation* = 0.96, *d* = 0, 95% CI [-0.05, 0.05]). Consistent with previous results[3], our study show that as task difficulty decreased (quantified as the absolute difference between the percentage of dots moving rightward and 50%), the assigned weights decreased as well (b = -0.28, $t(120)$ = -5.03, p < 0.001).

Participants were more confident when they interacted with the biased algorithm, as compared to the accurate algorithm (*M_Confidence* $_{\text{biased AI}}$ = 0.584, *M_Confidence* $_{\text{accurate AI}}$ = 0.558, *P permutation* = 0.003, *d* = 0.28, 95% CI [0.01, 0.04]) and noisy algorithm (*M_Confidence* $_{\text{noisy AI}}$ = 0.565, *P permutation* = 0.027, *d* = 0.20, 95% CI [0.002, 0.034]). No differences in confidence were found between the accurate and noisy algorithms (*P permutation* = 0.37, *d* = 0.08, 95% CI [-0.01, 0.03]), nor between any of the algorithms and baseline confidence (all *p*'s > 0.18).

Alternative analysis - Experiment 3

In Exp. 3 we examined changes in participants' classification of different demographic groups as financial managers after exposure to Stable Diffusion images. The results of this experiment showed a significant increase in the likelihood of choosing White men as financial managers. We used multinomial logistic regression, because the dependent variable involved choice from various unordered categories. Following a reviewer's request, we conducted an alternative analysis using permutation tests to compare the probability of selecting each demographic group before and after exposure to AI-generated images.

This alternative analysis confirmed our findings. The likelihood of choosing White men as financial managers increased significantly following exposure to AI-generated images (*P* permutation test = 0.01, *d* = 0.36, 95% CI [1.59, 13.5]; see **Fig. 3**). In contrast, no significant differences were observed for White women (*P* permutation test = 0.73, *d* = 0.05, 95% CI [-2.89, 4.09]), Asian men (*P* permutation test = 0.44, *d* = -0.11, 95% CI [-4.31, 1.93]), Asian women (*P* permutation test = 0.36, *d* = -0.13, 95% CI [-4.93, 1.93]) and Black men (*P* permutation test = 0.38, *d* = -0.13, 95% CI [-3.75, 1.3]). Notably, exposure to AI-generated images decreased the likelihood of selecting Black women as financial managers (*P* permutation test = 0.03, *d* = -0.30, 95% CI [-7.88, -0.47]).

In the control condition, where participants were exposed to neutral images, no significant differences emerged across groups (all *p*'s > 0.33), except for Black women, who were more likely to be chosen after exposure to the neutral images (*P* permutation test = 0.003, *d* = 0.43, 95% CI [0.01, 0.04]), possibly because participants corrected their initial biases.

Further, we compared the magnitude of change in participants' choices (between baseline and post-exposure responses) across the experimental and control groups for the different

demographic groups. Specifically, we assessed whether the increase in selecting 'White men' as financial managers in the experimental group was significantly greater than the changes observed for other demographic groups when compared to the control group.

The effect for White men was significantly larger than for White women (*P* permutation test = 0.03, *d* = 0.43, 95% CI [0.01, 0.14]), Asian women (*P* permutation test = 0.005, *d* = 0.54, 95% CI [0.03, 0.16]), Black men (*P* permutation test = 0.009, *d* = 0.51, 95% CI [0.02, 0.15]), and Black women (*P* permutation test = 0.001, *d* = 0.62, 95% CI [0.04, 0.17]). However, the difference was not statistically significant compared to Asian men (*P* permutation test = 0.06, *d* = 0.37, 95% CI [0, 0.13]). These results demonstrate that exposure to AI-generated images increased the bias in favor of selecting White men as financial managers.

Estimating Likelihood of Being a Financial Manager by Demographic Group

In Exp. 3, we presented participants six images of faces from different races and gender groups and asked: '*Who is most likely to be a financial manager?*'. Contrary to Experiments 1 and 2, in this task there is no objectively 'correct' answer, not least because race and gender are not necessarily good indicators of how likely a person is to be a financial manager. Given, however, that no further information was provided, participants may rely on race and gender to respond. That is, they may have answered the question: '(based on their race and gender) who is most likely to be a financial manager?'. The answer to this question depends on various factors, such as which country the financial manager works in, and so on. Thus, even for this question there is no definitive ground truth. Nevertheless, we show below that selecting 'White man' is likely not a normative response.

To address the question '*(based on race and gender) Who is most likely to be a financial manager?*', we estimate the probability of being a financial manager given a person's demographic group. We use U.S. as an example due to available statistics from the U.S. Bureau of Labor Statistics[4] (however the principal conclusion likely holds in many other countries. According to this data, among financial managers 44.3% are men and 55.7% are women. Additionally, 78.5% are white, 9.6% are Asian and 9.3% are Black. These percentages do not sum up to 100% because they do not represent all races. Therefore, we treated these groups as a whole, and approximate that among them about 80.6% are White, 9.85% are Asian and 9.55% are Black. Assuming equivalent distribution of men and women across racial group, we assessed that 35.71% of financial managers are White men, 44.89% are White women, 4.36% are Asian men, 5.49% are Asian women, 4.23% are Black men and 5.32% are Black women. Next, we examined the distribution of race and gender in the U.S.[5] which is 75.5% White, 6.3% Asian and 13.06% Black and 50.4% women. Therefore based on these demographic data, the probability of being a financial manager given a White man = (Percentage of financial managers that are white

men)/(Percentage of population that are white men) = (0.443·0.806·Total number of financial managers)/(0.755·0.496· U.S. population) = 0.95·Total number of financial managers/U.S. population. Doing similar calculations, the results for the other groups are: White women = 1.18·Total number of financial managers/U.S. population, Asian Men = 1.40·Total number of financial managers/U.S. population, Asian Women = 1.73·Total number of financial managers/U.S. population, Black Men = 0.65·Total number of financial managers/U.S. population and Black Women = 0.81·Total number of financial managers/U.S. population. Because the ratio "Total number of financial managers/U.S. population" remains constant across all demographic groups, the comparison focuses on the groups' coefficients. Among them, the coefficients of Asian women (1.73), Asian men (1.40) and White women (1.18) are greater than that of White men (0.95). Thus, based purely on demographic group, White men are unlikely to be a normative answer.

Supplementary models

A computational model suggests humans learn to be biased from a biased AI. Our tasks in Exp. 2 were designed such that a modulation in the participants' independent judgements indicated that they learned to become more like the algorithm, rather than just mimicking its response. We next use computational modelling to characterize this learning process. To that end, we fitted several reinforcement learning models[6,7] to the RDK data (Exp. 2). First, we examined the performance of a baseline model, which does not assume learning from the algorithms, defined as:

$$Response_t = b_0 + b_1 \cdot Evidence + \varepsilon_t$$

This model postulates that response is a noisy function of the evidence (i.e., the actual percentage of dots moving from left to right). It includes two free parameters: intercept (b_0) and slope (b_1), which map between the participant's internal estimation of the evidence and the external response scale. We compare this baseline model to the following learning model:

$$Response_t = b_0 + b_1 \cdot Evidence_t + Learned\ bias_t + \varepsilon_t$$

$$Learned\ bias_t = Learned\ bias_{t-1} + \alpha \cdot (Response\ AI_{t-1} - Response\ Participant_{t-1})$$

The learning model assumes that in addition to the evaluation of the evidence, a participant's response is also based on the exposure to past responses of the algorithm. In particular, the participant's response (i.e., estimation of the number of dots that move to the right) is assessed against the algorithm's response: If the response of the algorithm is higher than that of the participant (e.g., the algorithm indicates that 75% of dots move to the right, whereas the participant's response is 52%), the participant will tend to overestimate the percentage of right moving dots in the next trial. If, however, the response of the algorithm is lower than that of the participant (e.g., the algorithm indicates the 53% of dots move to the right, whereas the participant's response is 72%), the participant will underestimate the

percentage of right moving dots on the next trial. The model includes three parameters: intercept (b_0) and slope (b_1), the weight assigned to the learned bias (b_2) and learning rate parameter (α). The learned bias at t = 1 was set to 0.

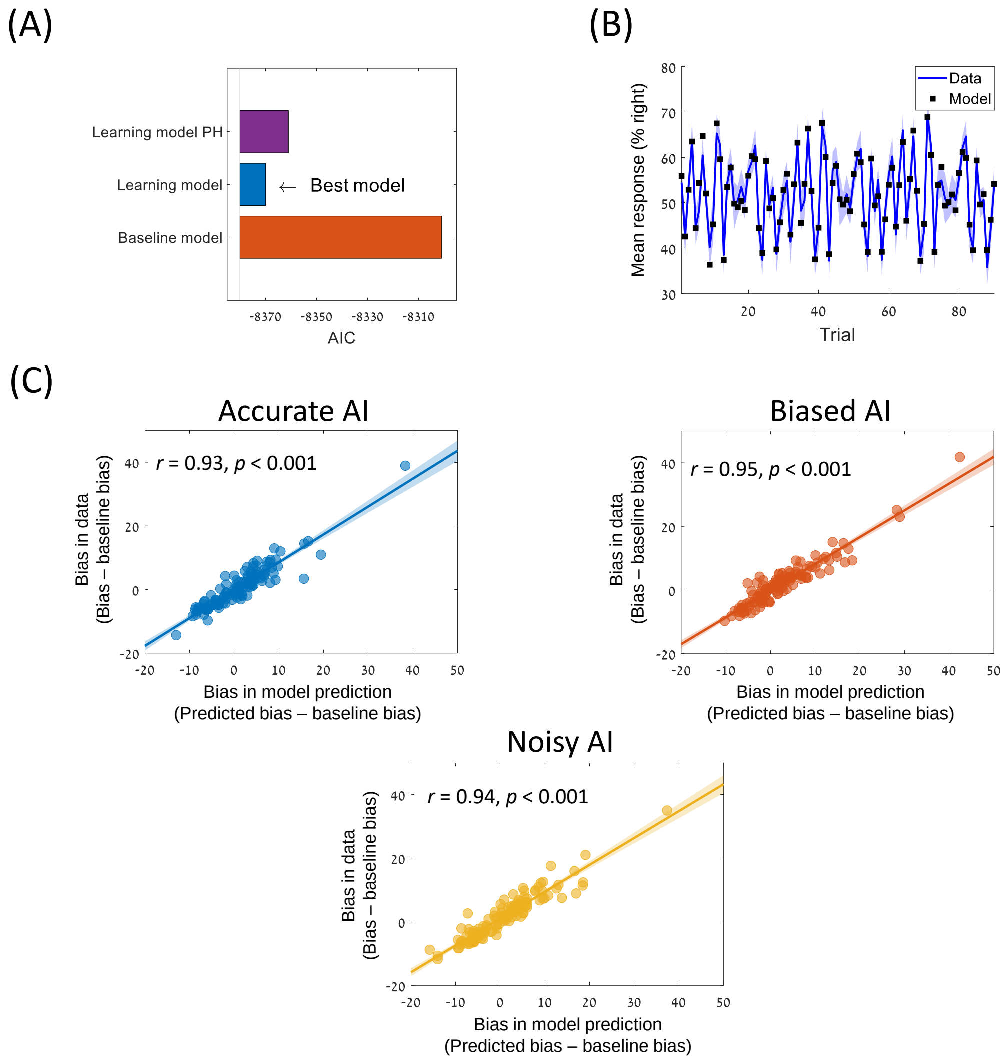

***Fig. S1. Computational modeling suggests humans learn to become biased**. (A) **Model comparison results**. Both learning models outperformed the baseline model, as indicated by their lower AIC scores. The simpler learning model outperforms the PH learning model. (B) **Model validation.** Predictions of the learning model using the best fit parameters (black dots) show a good fit to participants mean response (blue line) across trials. Blue shaded area corresponds to 95% confidence interval. (C) **Model predicted bias correlates with actual data across conditions.** Significant correlations between the bias levels predicted by the learning model (using the best fitted parameters) and the bias of the participants for the accurate (blue), biased (red) and noisy (yellow) algorithms. Each data point represents a single participant. Shaded areas correspond to 95% confidence intervals. All significant p-values remained significant after applying the Benjamini-Hochberg FDR correction at α = 0.05.*

In addition to these models, we tested another variant of the learning model assuming that learning rate is modulated by the absolute magnitude of the reward prediction error (hereafter PH learning model[8]). The Akaike Information Criterion (AIC[9]) scores of the models are presented in **Fig. S1A** (lower valued indicate a better fit). As shown, both learning models decisively outperform the baseline model. In addition, the PH learning model showed poorer fit than the simpler learning model. The best fitted parameters of the learning model were all significantly greater than 0: M_{b0} = 0.17, 95% CI [0.15, 0.20], M_{b1}= .66, 95% CI [0.62, 0.70], $M\alpha$ = 0.004, 95% CI [0.003, 0.005]. **Fig. S1B** shows the mean response of the participants across trials (blue line), which is well captured by the learning model (black dots). **Fig. S1C** demonstrates that the learning model (*x*-axis) accurately predicts the bias of the participants (*y*-axis, each point represents a single participant) when interacting with the accurate, biased and noisy algorithms.

The learning model suggests that humans learn to be biased over time. This model captures the tendency to produce biased responses while interacting with the biased algorithm, as compared to the accurate and noisy algorithms.

Supplementary experiments

Supplementary Exp. 1: Base rate of Response change

An additional experiment was conducted to investigate the base rate of response change in Exp. 1/Level 3. An overall of 50 participants took part in the experiment (consistent with the other conditions of Exp. 1). One outlier participant was excluded from analyses (z-score = 6.04, all results remained unchanged even when this participant was included).

The experiment followed a structure similar to that of Exp. 1. Participants first classified arrays of 12 emotional faces as either 'more sad' or 'more happy' in baseline blocks of 150 trials. Subsequently, they completed 300 trials of the same task but were given the option to change their initial decisions (by answering ‘Yes’ or ‘No’ when prompted). Importantly, unlike the other conditions of Exp. 1, here participants did not interact with any associate (neither AI or human). As in Exp. 1, participants were given a bonus payment based on their final decision, and thus were incentivized to change initial decisions which they considered to be errors.

Analysis of the data showed that the mean decision change rate was 3.97% (± 0.74% *SE*). This rate was significantly lower than in any of the interaction conditions in our study when there were disagreements (all p’s < 0.001). Moreover, it was significantly higher than the interaction conditions when there were agreements (all p’s < 0.001). The rate of decision change did not vary across blocks, as indicated by a linear mixed model predicting decision changes from block number as a fixed factor with random intercepts and slopes at the participant level (b = 0.001, $t(194)$ = 0.28, p = 0.77).

The low baseline change rate shows that participants rarely change their decisions on their own. Rather, interaction with an associate affects change rates. Specifically, agreement reinforces the initial decision, making changes even less likely, while disagreement increases decision changes compared to baseline rates.

Supplementary Exp. 2: Using Different Human Training Methods

To examine the generalizability of the method used to "train" human participants in Exp. 1/Level 2, we conducted a control experiment using an alternative approach. A new group of participants (N = 50) completed a modified protocol that involved actively predicting the responses of the participants from Level 1. Each correct prediction was awarded one point, which was converted to monetary reward at the end of the experiment, thereby incentivizing accurate predictions of other's judgements.

The results of this experiment were essentially consistent with the method used to train the human participants in Exp. 1/Level 2: participants characterized the arrays as 'more sad' 53.8% of the time, which is not different from chance (P permutation test against 50% = 0.08, d = 0.26, 95% $CI_{\text{'more sad'}}$ [50%, 58%]), and significantly lower than the AI algorithm (P permutation test < 0.001, d = 0.76, 95% CI [-0.07, -0.015]).

These findings demonstrate that the results are robust across different training methods, supporting the generalizability of our observations.

Supplementary Exp. 3: Equating Training Data Volume across Humans and AI

The human participants in Exp. 1/Level 2 were exposed to less data than the AI. To rule out the possibility that the difference between the bias amplification between the AI and humans at Level 2 is solely due to humans receiving less training labels, we repeated the same procedure with a new pool of participants (N = 50). However, this time we trained both the humans and the AI system (CNN) on the exact same subset of 200 arrays. The subset data were semi-randomly sampled from the original data set, preserving the bias and accuracy of the full set (with differences in bias and accuracy not exceeding 1%).

The results showed that the CNN characterized the arrays as 'more sad' 63.3% of the time, while humans did so only 54.4% of the time. This difference was significant (P permutation test < .001, d = 0.69, 95% CI [0.05, 0.13]). Moreover, the frequency of 'more sad' responses of the human participants who were trained on 200 trials was no different than that of a group of participants who were trained on only 100 trials (M_{100}= 54.8%, M_{200} = 54.4%, P permutation test = 0.93, d = 0.03, 95% CI [-0.04, 0.05]), suggesting that human learned bias does not increase with additional training examples.

Together, these findings demonstrate that the observed differences in bias amplification between AI and humans are unlikely to be driven by differences in the training samples sizes.

Supplementary Exp. 4: AI induced bias as a function of the bias magnitude

The results of Exp. 2 showed that human participants became more biased when interacting with a biased algorithm. Here, we examine the association between the magnitude of the bias exhibited by the AI algorithm and the bias induced by it. To this end, we used the paradigm employed in Exp. 2, with the following exceptions: i) Participants interacted only with a bias algorithm, ii) Participants performed two baseline blocks and three interaction blocks with a biased AI, each of which consisted of 20 trials. The percentage of the dots that moved rightward were: 6%, 16%, 22%, 24%, 28%, 32%, 36%, 40%, 44%, 48%, 52%, 56%, 60%, 64%, 68%, 72%, 76%, 78%, 86%, 96% (presented in a random order).

Three groups of participants (N_1 = 127, N_2 = 114, N_3 = 145) interacted with three different biased algorithms with average biases of 5.3 (small), 14.5 (medium) and 24.1 (large), respectively (each group interacted only with one algorithm). **Fig. S2A** shows the AI induced bias as a function of the magnitude of bias (small, medium and large). One samples *t*-tests against 0, revealed that the AI induced bias was significantly higher than 0 for all groups: low (M_{bias} = 1.34, $t(126) = 2.38$, $p = 0.019$), medium (M_{bias} = 3.06, $t(113) = 5.34$, $p < 0.001$) and high (M_{bias} = 3.65, $t(144) = 5.95$, $p < 0.001$).

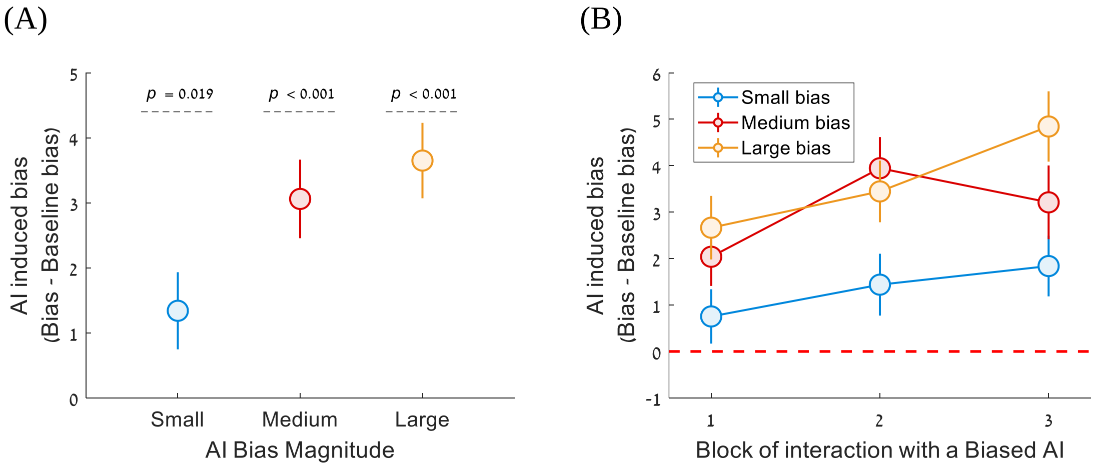

Fig. S2. *AI induced bias as a function of the bias magnitude (N = 386). (A) The AI induced bias was significantly higher than 0 for all levels of the AI magnitude bias (small, medium and large). P-values were derived using one-sample t-tests. All significant p-values remained significant after applying the Benjamini-Hochberg FDR correction at α = 0.05. (B) AI induced bias as a function of AI bias magnitude and block. The AI induced bias increased as a function of block. Error bars correspond to standard error of the mean.*

Fig. S2B further shows the AI induced bias of each group as a function of bias magnitude and block number. A two-way ANOVA with the bias magnitude (small, medium, large) and Block number (1, 2, 3) as independent variables and AI induced bias as a dependent variable, revealed a main-effect for bias magnitude, $F(2, 383) = 4.23$, $p = 0.015$. Follow-up post-hoc tests, indicate that the AI induced bias was higher when bias magnitude was large compared to small ($p = 0.01$). All other effects did not reach statistical significance

(all p's > 0.09). A main-effect was found also for block, $F(1.8, 690.27) = 9.78$, $p < 0.001$. Follow-up post-hoc tests, showed that the AI induced biased was higher in the third ($p < 0.001$) and second ($p < 0.001$) blocks as compared to the first one. No difference was found between the second and third blocks ($p = 0.309$). The interaction between bias magnitude and block number did not reach a statistical significance, $F(3.6, 690.27) = 1.79$, $p = 0.135$.

These results replicate the AI induced bias effect shown in Exp. 2. The results also replicate the increase of the AI induced bias across blocks. Moreover, the results show that even if the bias of the AI is as low as 5%, the participants are still significantly influenced by it.

Supplementary Experiment 5: AI induced bias in social judgements

In the main text, we demonstrated that interacting with biased AI systems can amplify human biases in perceptual, emotional and social tasks. This supplementary experiment aimed to provide further support for AI induced bias in social judgements, specifically examining whether interaction with a gender-biased AI algorithm could influence human judgements. This context is particularly relevant given the increasing use of AI in hiring and professional evaluation processes[10].

Unlike the emotion aggregation (Exp. 1) and moving dots (Exp. 2) tasks, participants in this experiment were likely to be aware that we were assessing gender bias. Thus, they might attempt to correct their responses to avoid displaying such bias. To address this potential problem, we designed a two-step task. The first part included direct comparisons between men and women, where biases are relatively apparent, while the second part included separate evaluations of men and women, where biases are less apparent[11]. We assumed that in the direct comparison task, participants would be more aware of the potential gender bias and attempt to suppress it. However, bias induced by the algorithm in this part may carry over and emerge in the separate evaluation task, where task structure makes biases less apparent and thus less likely to be suppressed.

As in Exps. 1-3 in the main text, participants' baseline performance was measured prior to interacting with the algorithm ($N = 45$). The baseline phase consisted of two parts. First, participants completed 45 trials in a 'relative evaluation' task (**Fig. S3A**). Participants were informed that one key skill required for architecture studies is the ability to accurately copy shapes, and that they would be asked to evaluate applicants based on this ability. On each trial, a simple geometrical shape was presented on screen for one second, alongside photos of two applicants and their attempt to copy it. Participants rated which applicant copied the shape more accurately on a scale ranging from one applicant to the other (self-paced; **Fig. S3A**). Subsequently, participants completed the 'separate evaluation' task (**Fig. S3B**), in which they were presented with images of all applicants one-by-one and asked to estimate each applicant's likelihood of succeeding in architecture studies on a scale ranging from 'Not at all' (coded as 1) to 'Very much' (coded as 7). This task was self-paced and consisted of 10 trials.

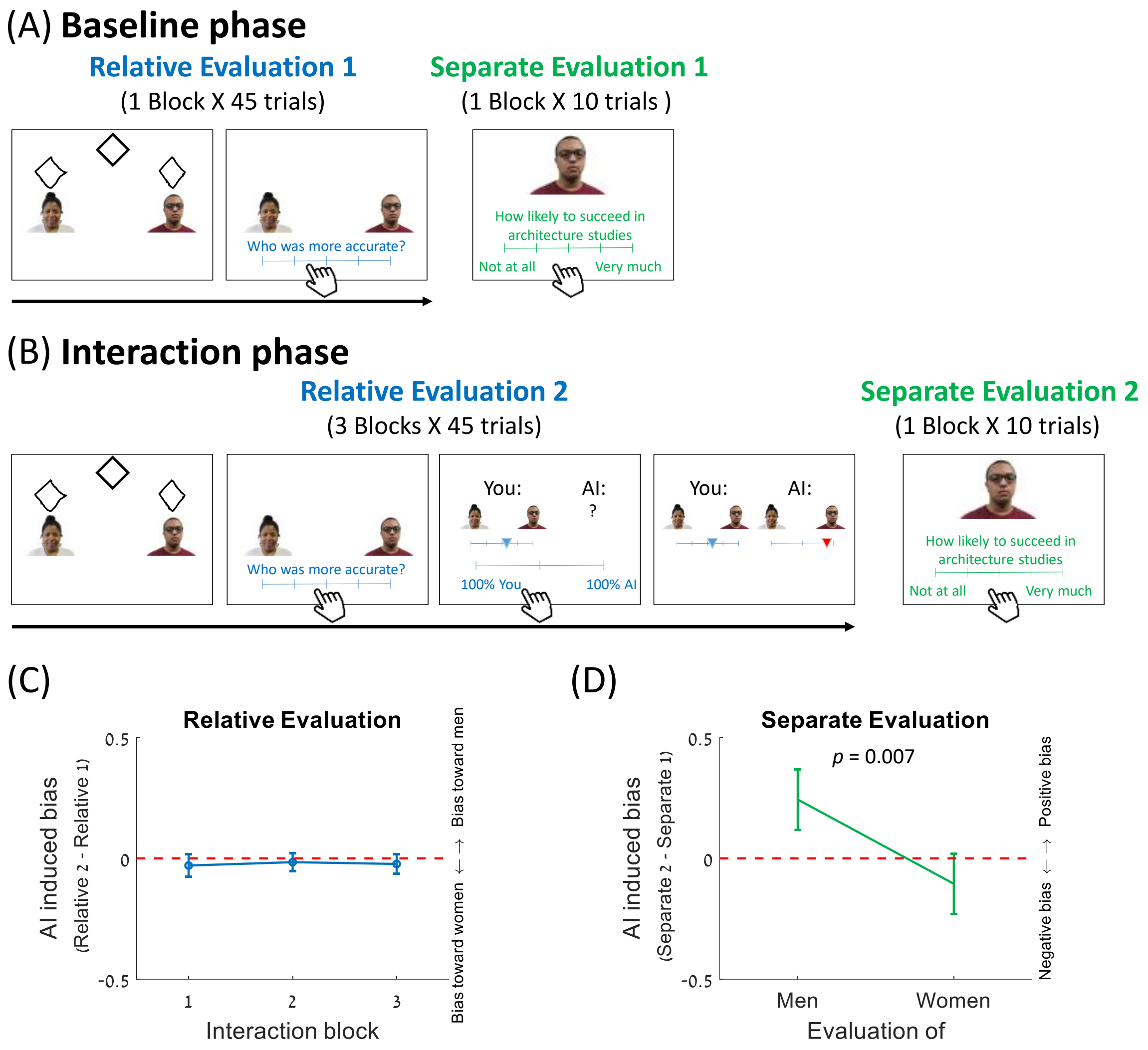

Fig. S3. Gender biased algorithm induces gender bias in humans**. (N = 45). (A) **Baseline Phase**. Participants were told that a key skill required for architecture studies is the ability to accurately copy shapes. On each of 45 trials, a simple geometrical shape was presented to them, next to photos of two applicants and their attempt to copy it (1 sec). The participants estimated which applicant copied the shape more accurately (relative evaluation, self-paced). Second, the participants were presented with images of all 10 applicants one-by-one and estimated their likelihood to succeed in architecture studies (separate evaluation, self-paced). (B) **AI-Interaction Phase**. Participants first performed the same procedure as in the baseline blocks using the same set of photos. They were then asked to assign weights to their response and the response of the algorithm on a scale ranging from '100% You' to '100% AI' to determine the final joint decision (self-paced). Thereafter, they were presented with the algorithm's response (2 sec), which was accurate for same gender trials but biased towards men otherwise (relative evaluation). Participants then evaluated the applicant one-by-one, exactly as in the baseline phase (separate evaluation, self-paced). (C) **No AI induced bias in the relative evaluation task**. Positive values indicate bias toward men after interacting with the AI and negative values indicate bias toward woman. (D) **AI induced bias in the separate

***evaluation task**, such that participants evaluated men as more competent after interacting with the AI relative to before, with no change for evaluation of women. Error bars correspond to standard error of the mean. Face stimuli in panel (A) are adapted from ref. [12] under a Creative Commons licence CC BY 4.0.*

We used photos of 10 applicants – five men and five women – taken from the American Multiracial Faces Database[12]. Participants completed 45 trials in which they were presented with all possible combinations of pairs of applicants. The original shapes used included a square, a square rotated by 45 degrees, a 4-point star rotated by 45 degrees, a heart, and arrows pointing up, down, right and left. The copied shapes were created for the current experiment by manually copying the original shapes using a computer mouse. The total surface area of the original and copied shapes was identical.

Following the baseline phase, participants proceeded to the interaction phase. This phase mirrored the structure of the baseline phase but included interaction with an AI algorithm (**Fig. S3B**). After indicating their initial judgement, participants assigned weights to their judgement and to that of the algorithm on a scale ranging from '100% You' to '100% AI' to determine the final joint decision. Following this, they were presented with the algorithm's response for 2 seconds (**Fig. S3B**). The algorithm's responses were designed to be accurate in same-gender trials (i.e., both applicants were men, or both were women) but biased towards men in mixed-gender trials (i.e., a man and a woman applicants).

The accuracy of the copied shapes was quantified by calculating the correlation between the copied and original images. A correlation of 1 indicated that the two images completely overlap, with higher differences between the shapes resulting in lower correlation between them. The copied shapes of men and women did not differ in accuracy ($r_{men} = r_{women} = 0.83$). The algorithm's biased responses were created by overestimating the accuracy of the man applicant in the 'mixed gender' trials (*Mean bias* = 0.3, *SD* = 0.15, range of 0.1-0.42). Before interacting with the AI, participants were told that they "will be presented with the response of an AI algorithm that was trained to perform the task". The participants performed 135 trials divided into 3 blocks. In each block all the possible combinations of pairs of applicants were presented.

The results revealed that participants' separate evaluations of men applicants increased following the interaction with the biased algorithm (4.42 ± 0.10, *M* ± *SE*) relative to baseline (4.26 ± 0.08, *M* ± *SE*; see **Fig. S4D**), this increase was significantly greater than zero (*P* permutation test = 0.028, *d* = 0.34, 95% CI [0.04, 0.45]). The increase in evaluation of men was significantly greater than the non-significant change in the evaluation of women (*P* permutation test = 0.007, *d* = 0.41, 95% CI [0.11, 0.60]). As predicted, the relative evaluations remained steady (*P* permutation test against 0 = 0.55, *d* = -0.09, 95% CI [-0.10, 0.04]), with no difference found after (compared to before) interacting with the algorithm (**Fig. S4C**).

These results indicate that interacting with a biased algorithm altered participants' evaluations. In particular, participants became more biased in gender-based judgements after interacting with the algorithm, perceiving men applicants as more likely to succeed. The findings also imply that participants were able to suppress the effect of bias amplification when it was explicitly apparent, but not when it was less obvious.

Supplementary Exp. 6: Replication & Extension of Exp. 3 (Stable Diffusion).

The present experiment aimed to replicate and extend the findings of Exp. 3. A total of 200 participants took part in the study ($N_{\text{AI group}} = 91$, $N_{\text{Control group}} = 109$). Participants in the AI group were instructed to envision themselves creating a presentation about financial managers. They were given a definition of the role and instructed to choose an image of a financial manager for their presentation in each trial. Critically, they were explicitly informed that the images of the financial managers were generated by Stable Diffusion and were given the precise prompt used to generate them ("A color photo of a financial manager, headshot, high-quality"). Conversely, participants in the control group received similar instructions but were tasked with creating a presentation on fractals instead. No time constraints for image selection were imposed on either group.

Following this stage, both groups were presented with images of six individuals: a White man, a White woman, an Asian man, an Asian woman, a Black man and a Black woman (an in Exp. 3 – stage 3). They were then asked: 'which person is most likely to be a financial manager?'. The stimuli remained consistent with those used in Exp. 3. Participants were reminded of the subjective nature of the task and encouraged to answer based on their genuine preferences. The order of the trials was randomized for all the stages across participants.

Consistent with Exp. 3, the data of this study was analyzed using a mixed-model multinomial logistic regression with the type of images participant were exposed to (AI images vs. Control images) as a fixed factor with random intercepts and slopes at the participant level.

The findings replicated those from Exp. 3 (see **Fig. 3B**), showing a significant increase in participants' tendency to choose White Men as financial managers following the active interaction with the AI-generated images compared to the control images, $F(5, 246) = 3.63$, $p = 0.003$ (**Fig. S4**). Specifically, it was found that compared to exposure to the control images, exposure to AI images increased the likelihood of choosing White men as financial managers (**Fig. S4**, blue markers) compared to Asian men ($b = 0.36$, $t = 2.04$, $p = 0.04$, 95% CI [0.013, 0.71]), Asian women ($b = 0.36$, $t = 2.10$, $p = 0.03$, 95% CI [0.02, 0.72]), Black men ($b = 0.51$, $t = 2.07$, $p = 0.04$, 95% CI [0.02, 0.99]) and Black women ($b = 0.60$, $t = 2.32$, $p = 0.02$, 95% CI [0.09, 1.12]). A significant difference was not found compared to White women ($b = 0.22$, $t = 1.30$, $p = 0.19$, 95% CI [-0.11, 0.56]).

For robustness the data was also analyzed using non-parametric permutation tests. Consistent with the previous analysis, the likelihood of choosing White men as financial managers significantly increased following the interaction with the AI-generated images compared to the control images (*P* permutation test = 0.01, *d* = 0.36, 95% CI [1.59, 13.5]; **Fig. S4**). As expected, no significant difference was observed for White women (*P* permutation test = 0.73, *d* = 0.05, 95% CI [-2.89, 4.09]), Asian men (*P* permutation test = 0.44, *d* = -0.11, 95% CI [-4.31, 1.93]), Asian women (*P* permutation test = 0.36, *d* = -0.13, 95% CI [-4.93, 1.93]) and Black men (*P* permutation test = 0.38, *d* = -0.13, 95% CI [-3.75, 1.3]). Moreover, following the interaction with the AI-images, there was a decreased likelihood of choosing Black women as financial managers (*P* permutation test = 0.03, *d* = -0.30, 95% CI [-7.88, -0.47]).

These results replicate and extend the results of Exp. 3, demonstrating that active engagement with the biased AI-images (Stable Diffusion) alter participants' internal representation of financial managers, highlighting how interaction with biased AI systems can amplify societal biases.

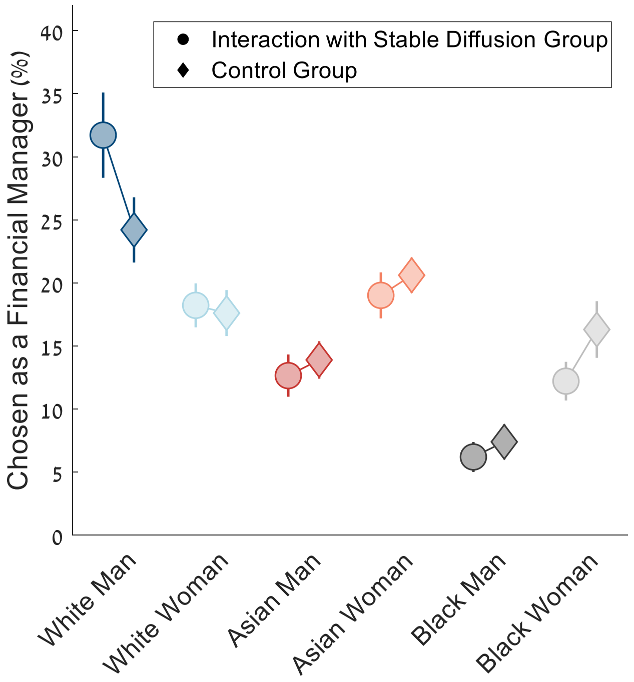

Fig. S4. Active engagement with Stable Diffusion images amplifies human bias (N = 200). *The results of Supplementary Exp. 6 reveal a significant increase in the tendency of participants to choose White men as financial managers following the active interaction with the AI-generated images compared to the control images. Conversely, no significant differences emerged for White women, Asian men, Asian women and Black men. Moreover, the results show that following the interaction with the AI-images, there was a decreased likelihood of choosing Black women as financial managers.*

References

1. Haberman, J., Harp, T. & Whitney, D. Averaging facial expression over time. *J Vis* **9**, (2009).
2. He, K., Zhang, X., Ren, S. & Sun, J. Deep residual learning for image recognition. in *Proceedings of the IEEE Computer Society Conference on Computer Vision and Pattern Recognition* vols 2016-December (2016).
3. Liang, G., Sloane, J. F., Donkin, C. & Newell, B. R. Adapting to the algorithm: how accuracy comparisons promote the use of a decision aid. *Cogn Res Princ Implic* **7**, (2022).
4. U.S. Bureau of Labor Statistics. Current Employment Statistics Highlights. *https://www.bls.gov/cps/aa2022/cpsaat11.htm* (2022).
5. United States Census Bureau. *https://www.census.gov/quickfacts/fact/table/US/PST045219* (2022).
6. Huys, Q. J. M. *et al.* Bonsai trees in your head: How the pavlovian system sculpts goal-directed choices by pruning decision trees. *PLoS Comput Biol* **8**, (2012).
7. Niv, Y. Reinforcement learning in the brain. *J Math Psychol* **53**, (2009).
8. Pearce, J. M. & Hall, G. A model for Pavlovian learning: Variations in the effectiveness of conditioned but not of unconditioned stimuli. *Psychol Rev* **87**, (1980).
9. Akaike, H. A New Look at the Statistical Model Identification. *IEEE Trans Automat Contr* **19**, (1974).
10. Dastin, J. Amazon Scraps Secret AI Recruiting Tool that Showed Bias against Women. in *Ethics of Data and Analytics* (2022). doi:10.1201/9781003278290-44.
11. Crandall, C. S. & Eshleman, A. A Justification-suppression Model of the Expression and Experience of Prejudice. *Psychological Bulletin* vol. 129 Preprint at https://doi.org/10.1037/0033-2909.129.3.414 (2003).
12. Chen, J. M., Norman, J. B. & Nam, Y. Broadening the stimulus set: Introducing the American Multiracial Faces Database. *Behav Res Methods* **53**, (2021).
