# Supplementary material for: How human–AI feedback loops alter human perceptual, emotional and social judgements
Source: Nat Hum Behav. 2024 Dec 18;9(2):345–59. doi: 10.1038/s41562-024-02077-2 (PMC11860214; doi:10.1038/s41562-024-02077-2)
Supplement: Supplementary file 2 — Reporting Summary [file 41562_2024_2077_MOESM2_ESM.pdf]

nature portfolio

Corresponding author(s): Moshe Glickman & Tali Sharot

Last updated by author(s): Oct 19, 2024

Reporting Summary

Nature Portfolio wishes to improve the reproducibility of the work that we publish. This form provides structure for consistency and transparency in reporting. For further information on Nature Portfolio policies, see our Editorial Policies and the Editorial Policy Checklist.

Statistics

For all statistical analyses, confirm that the following items are present in the figure legend, table legend, main text, or Methods section.

n/a	Confirmed	
☐	☒	The exact sample size (*n*) for each experimental group/condition, given as a discrete number and unit of measurement
☐	☒	A statement on whether measurements were taken from distinct samples or whether the same sample was measured repeatedly
☐	☒	The statistical test(s) used AND whether they are one- or two-sided<br>*Only common tests should be described solely by name; describe more complex techniques in the Methods section.*
☐	☒	A description of all covariates tested
☐	☒	A description of any assumptions or corrections, such as tests of normality and adjustment for multiple comparisons
☐	☒	A full description of the statistical parameters including central tendency (e.g. means) or other basic estimates (e.g. regression coefficient) AND variation (e.g. standard deviation) or associated estimates of uncertainty (e.g. confidence intervals)
☐	☒	For null hypothesis testing, the test statistic (e.g. *F*, *t*, *r*) with confidence intervals, effect sizes, degrees of freedom and *P* value noted<br>*Give P values as exact values whenever suitable.*
☒	☐	For Bayesian analysis, information on the choice of priors and Markov chain Monte Carlo settings
☐	☒	For hierarchical and complex designs, identification of the appropriate level for tests and full reporting of outcomes
☐	☒	Estimates of effect sizes (e.g. Cohen's *d*, Pearson's *r*), indicating how they were calculated

Our web collection on statistics for biologists contains articles on many of the points above.

Software and code

Policy information about availability of computer code

Data collection	Data were collected online. Experiments were designed in PsychoPy3 (2022.2.5) and hosted on the Pavlovia platform.
Data analysis	Matlab R2017b (Mathworks, Inc.), Google Colab notebook (Python 3.10), IBM SPSS 27.<br>Code is available at: https://github.com/affective-brain-lab/BiasedHumanAI

For manuscripts utilizing custom algorithms or software that are central to the research but not yet described in published literature, software must be made available to editors and reviewers. We strongly encourage code deposition in a community repository (e.g. GitHub). See the Nature Portfolio guidelines for submitting code & software for further information.

Data

Policy information about availability of data

All manuscripts must include a data availability statement. This statement should provide the following information, where applicable:

- Accession codes, unique identifiers, or web links for publicly available datasets
- A description of any restrictions on data availability
- For clinical datasets or third party data, please ensure that the statement adheres to our policy

Data are available at: https://github.com/affectivebrain-lab/BiasedHumanAI
Chicago Face Database, used in Experiment 3 & Supplementary Experiment 6, available at: https://chicagofaces.org/.
American Multiracial Faces Database, used in Supplementary Experiment 5, available at: https://jacquelinemchen.wixsite.com/sciplab/face-database

Research involving human participants, their data, or biological material

Policy information about studies with human participants or human data. See also policy information about sex, gender (identity/presentation), and sexual orientation and race, ethnicity and racism.

Reporting on sex and gender	Participants self-reported their gender (man/woman/other). No gender based analyses were conducted, as our main interest was how biased AI systems influence humans in general, rather than whether gender modulates the effect.
Reporting on race, ethnicity, or other socially relevant groupings	See above.
Population characteristics	See Research sample.
Recruitment	Participants were recruited via Prolific (https://prolific.ac/) and received a payment of £7.5 per hour in exchange for participation, as well as a bonus fee ranging from £0.5 to £2.<br>A potential bias in the current study is self-selection bias. Since participants were recruited through an online platform (Prolific), there is a possibility that individuals with a higher interest in AI technologies may have been more willing to join the study. To mitigate this potential bias, the study was advertised as focusing on judgment and decision-making experiment, without specific mention of AI technologies.
Ethics oversight	All experiments were approved by the UCL Ethics Committee (3990/003 and EP_2023_013).

Note that full information on the approval of the study protocol must also be provided in the manuscript.

Field-specific reporting

Please select the one below that is the best fit for your research. If you are not sure, read the appropriate sections before making your selection.

☐ Life sciences ☒ Behavioural & social sciences ☐ Ecological, evolutionary & environmental sciences

For a reference copy of the document with all sections, see nature.com/documents/nr-reporting-summary-flat.pdf

Behavioural & social sciences study design

All studies must disclose on these points even when the disclosure is negative.

Study description	The study consisted of a series of experiments designed to investigate how human-AI interactions impact human judgments. Each experiment included a judgment task (e.g., emotion aggregation or social judgment), where participants made individual judgments either before or after interacting with an AI system. In the focal conditions, the participants were provided with feedback from the AI system to assess whether and how their judgments would change after receiving it.<br>The design involved both within-subjects and between-subjects factors, depending on the specific experiment. The within-subject factor typically involved collecting responses before and after the interaction with the AI, while between-subjects factors varied across different conditions (e. g., interaction with AI vs. human).
Research sample	A total of 1,401 individuals participated in this study. Sample sizes were determined based on pilot studies, and designed to achieve a statistical power of 0.8 (α = 0.05). For each experiment, the largest sample size required to detect the key effect of interest was used and rounded up to ensure sufficient power.<br>Experiment 1 – Level 1: N = 50 (32 women, 18 men, Mage = 38.74 ± 11.17 SD), experiment 1 – Human-Human – Level 2: N = 50 (23 women, 25 men, 2 not reported, Mage = 34.58 ± 11.87 SD), experiment 1 – Human-AI – Level 3: N = 50 (24 women, 24 men, 2 not reported, Mage = 39.85 ± 14.29 SD), experiment 1 – Human-Human – Level 3: N = 50 (20 women, 30 men, Mage = 40.16 ± 13.45 SD), experiment 1 – Human-AI-perceived-as-human – Level 3: N = 50 (15 women, 30 men, 4 not reported, 1 non-binary, Mage = 40.16 ± 13.45 SD), experiment 1 – Human-Human-perceived-as-AI – Level 3: N = 50 (18 women, 30 men, 1 not reported, 1 non-binary, Mage = 34.79 ± 10.80 SD), experiment 2: N = 120 (57 women, 60 men, 1 other, 2 not reported, Mage = 38.67 ± 13.19 SD), experiment 2 accurate algorithm: N = 50 (23 women, 27 men, Mage = 36.74 ± 13.45 SD), experiment 2 biased algorithm: N = 50 (26 women, 23 men, 1 not reported, Mage = 34.91 ± 8.87 SD), experiment 3: N = 100 (40 women, 56 men, 4 not reported, Mage = 30.71 ± 12.07 SD), Supplementary experiment 1: N = 50 (26 women, 17 men, 7 not reported, Mage = 39.18 ± 14.01 SD), Supplementary experiment 2: N = 50 (24 women, 23 men, 1 other, 2 not reported, Mage = 36.45 ± 12.97 SD), Supplementary experiment 3: N = 50 (20 women, 29 men, 1 not reported, Mage = 32.05 ± 10.08 SD), Supplementary experiment 4: N = 386 (241 women, 122 men, 7 other, 16 not reported, Mage = 28.07 ± 4.65 SD), Supplementary experiment 5: N = 45 (19 women, 23 men, 1 other, 2 not reported, Mage = 39.50 ± 14.55 SD), Supplementary experiment 6: N = 200 (85 women, 98 men, 5 other, 12 not reported, Mage = 30.87 ± 10.26 SD) and Supplementary experiment 6: N = 200 (85 women, 98 men, 5 other, 12 not reported, Mage = 30.87 ± 10.26 SD).<br>Samples were not representative.
Sampling strategy	A convenience sampling method was used, with participants recruited through Prolific, an online platform that allows individuals to voluntarily participate in experiments. Sample sizes were determined based on pilot studies, and designed to achieve a statistical power of 0.8 (α = 0.05). For each experiment, the largest sample size required to detect the key effect of interest was used and

	rounded up to ensure sufficient power.
Data collection	Data was collected using an online computerized behavioral tasks. Participants were recruited via Prolific (https://prolific.ac/) and received a payment of £7.5 per hour in exchange for participation, as well as a bonus fee ranging from £0.5 to £2. All participants had normal or corrected-to-normal vision. Since the study was conducted online, blinding the researcher to the experimental hypothesis was not relevant.
Timing	April 2021 to March 2024.
Data exclusions	In one experiment (Exp. 1: Human-Human Interaction/Level 2), 14 participants (out of 64) were excluded based on pre-established criteria. Specifically, participants were excluded if they gave incorrect answers on more than 10% of the trials, which indicated that they were not attending to the task. Including these participants did not change the results, and they were only excluded to rule out the possibility that the lack of bias amplification in the human-human condition was due to participants inattention.
Non-participation	No participants had dropped out or declined participation.
Randomization	Studies were conducted within-subject. Experimental conditions were inter-mixed.

Reporting for specific materials, systems and methods

We require information from authors about some types of materials, experimental systems and methods used in many studies. Here, indicate whether each material, system or method listed is relevant to your study. If you are not sure if a list item applies to your research, read the appropriate section before selecting a response.

Materials & experimental systems

n/a	Involved in the study	
☒	☐	Antibodies
☒	☐	Eukaryotic cell lines
☒	☐	Palaeontology and archaeology
☒	☐	Animals and other organisms
☒	☐	Clinical data
☒	☐	Dual use research of concern
☐	☐	Plants

Methods

n/a	Involved in the study	
☒	☐	ChIP-seq
☒	☐	Flow cytometry
☒	☐	MRI-based neuroimaging
